# All-atom simulations reveal distinct pathways for α_IIb_β_3_ activation by biochemical vs. mechanical cues

**DOI:** 10.1007/s00018-026-06138-9

**Published:** 2026-03-06

**Authors:** Reza Kolasangiani, Onkar Joshi, Martin A. Schwartz, Tamara C. Bidone

**Affiliations:** 1 Department of Biomedical Engineering, University of Utah, Salt Lake City, UT USA; 2 Scientific Computing and Imaging Institute, University of Utah, Salt Lake City, UT USA; 3 Department of Internal Medicine (Cardiology), Yale Cardiovascular Research Center, Yale University, New Haven, CT USA; 4 Department of Cell Biology, Yale University, New Haven, CT USA; 5 Department of Biomedical Engineering, School of Engineering and Applied Science, Yale University, New Haven, CT USA; 6 Department of Biochemistry, University of Utah, Salt Lake City, UT USA; 7 Department of Molecular Pharmaceutics, University of Utah, Salt Lake City, UT USA

**Keywords:** Integrins, Molecular conformational changes, Protein structures and functions, Molecular dynamics

## Abstract

The conformational activation of α_IIb_β_3_integrin is crucial for platelet aggregation, a central event in hemostasis and thrombosis. Although activation can be triggered by extracellular arginine-glycine-aspartic acid (RGD)-containing ligands as well as mechanical forces, how these biochemical and mechanical cues exactly govern the structural dynamics of α_IIb_β_3_remains unclear. Here, using all-atom molecular dynamics simulations, we show that mechanical force and RGD binding promote activation α_IIb_β_3_through distinct mechanisms. Mechanical force applied to the RGD-binding site induces long-range, correlated motions of distant parts of the receptor, facilitating head–leg separation. In contrast, RGD binding increases localized, non-correlated fluctuations that weaken leg coordination but do not generate long-range motions. Despite these differences, both cues stabilize the open, extended conformation of α_IIb_β_3_. Together, these findings suggest that mechanical and biochemical stimuli play complementary yet distinct roles in integrin conformational activation. A balance between global coordination and local fluctuations likely governs integrin activation in complex environments where the dominance of mechanical or biochemical cues could lead to distinct activation pathways and functional outcomes.

**Supplementary Information:**

The online version contains supplementary material available at 10.1007/s00018-026-06138-9.

## Introduction

**Fig. 1:**
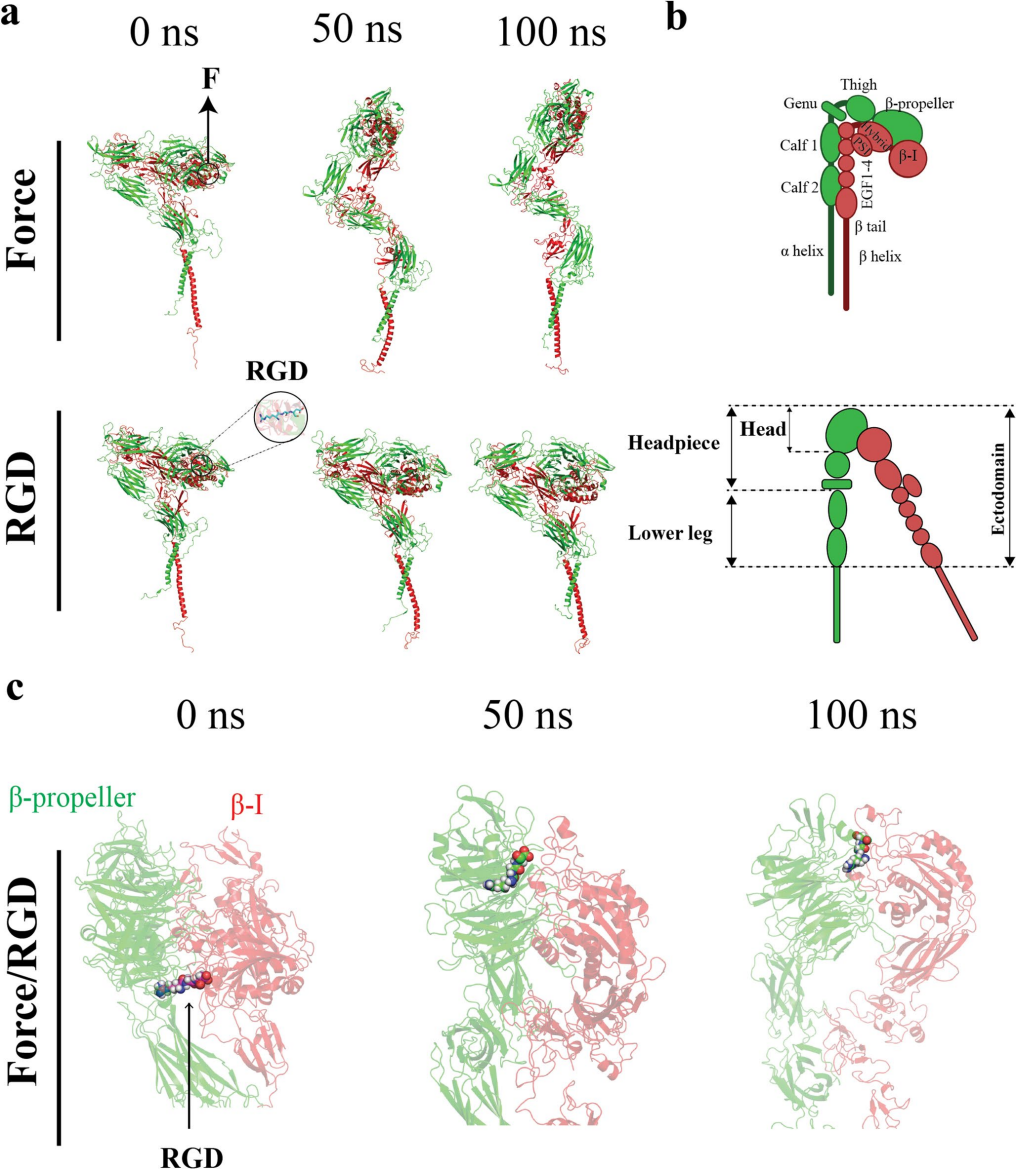
Structural organization and stimulus-dependent conformational dynamics of α_IIb_β_3_ from all-atom MD simulations. (**A**) All-atom secondary structure representations of α_IIb_β_3_ w: (top) subjected to mechanical force during SMD simulations, and (bottom) in complex with a bound RGD motif during equilibrium MD simulations. Structures are shown at 0, 50, and 100 ns. (**B**) Schematic representation of integrin in its bent/closed (top) and extended/open (bottom) conformations. The α_IIb_ subunit (green) includes β-propeller, thigh domain, and two calf domains, while the β_3_ subunit (red) consists of β-I domain, followed by the hybrid domain, the plexin-semaphorin-integrin (PSI) domain, four cysteine-rich epidermal growth factor (EGF) modules (I-EGF 1–4), and the β-tail domain (BTD). (**C**) All-atom secondary structure representations of α_IIb_β_3_ upper ectodomain under the Force/RGD condition at 0, 50, and 100 ns. The RGD is shown in CPK representation (carbon = cyan, oxygen = red, nitrogen = blue, hydrogen = white)

Platelet activation is essential for normal hemostasis, protecting against excessive blood loss following vascular injury. A central mediator of this process is integrin α_IIb_β_3_, the primary platelet receptor for fibrinogen and fibrin [1, 2]. In circulating platelets, α_IIb_β_3_ adopts a bent, closed conformation with low ligand bibnding affinity. Upon platelet activation, α_IIb_β_3_ undergoes a transition to an extended, open conformation with high ligand binding affinity. This process must be tightly regulated: insufficient α_IIb_β_3_ activation leads to bleeding disorders such as Glanzmann’s thrombasthenia, whereas excessive activation drives pathological platelet aggregation and thrombosis [3–7]. Structurally, α_IIb_β_3_ is a noncovalent heterodimer whose extracellular region, or ectodomain, comprises the headpiece—formed by the α_IIb_ β-propeller and thigh domains and the β_3_ βI and hybrid domains—and two lower legs composed of the α_IIb_ calf domains and the β_3_ PSI, EGF 1–4, and β-tail domains (Fig. 1A, B) [8, 9]. Repositioning of these domains underlie the transition from bent to extended states.

Multiple cues can trigger this conformational transition in the vasculature. These include ariginine-glycine-aspartic acid (RGD)-containing ligands such as fibrinogen, fibrin, and fibronectin [10–18]. Though, resting platelets can adhere to fibrin clots or immobilized fibrinogen, maintaining function for the low-affinity state of integrin when the ligand resists force [19]. Under shear stresses—ranging from < 1,000 s⁻¹ in veins and large arteries [20–23] to > 1,000 s⁻¹ in arterioles, microvessels, or stenotic regions [24–28]— α_IIb_β_3_ must withstand force while intracellular tension is applied to the β_3_ cytoplasmic region.

In addition to ligand binding and mechanical force, α_IIb_β_3_ activation can also be induced by divalent cations such as Mn^2+^ and Mg^2+^ [29, 30] or specific antibodies [31–34], even in the absence of canonical RGD motifs [35, 36]. Despite extensive structural and biophysical studies of α_IIb_β_3_, a central question remains unresolved: do mechanical and biochemical stimuli activate α_IIb_β_3_ through shared or distinct structural pathways? Addressing this question has proven challenging as most approaches analyze multiple pathways simultaneously, making it difficult to isolate cue-specific effects on integrin structural dynamics.

To overcome this limitation, we employed all-atom molecular dynamics simulations and network anlaysis. We show that force activates the receptor by propagating long-range, highly correlated motions across the ectodomain, culminating in head–leg separation. In contrast, RGD binding elicits localized fluctuations in the β3 transmembrane helix, weakening interdomain coordination rather than promoting global structural reorganization. Yet, despite these differences, both stimuli stabilize the extended, open conformation characteristic of activated α_IIb_β_3_. Together, these findings demonstrate that α_IIb_β_3_ activation does proceed through a single, unified structural pathway. Instead, biochemical and mechanical cues act through complementary but distinct routes. In the complex environment of the vasculature, the balance between global coordination and localized fluctuations of α_IIb_β_3_ may determine the dominant activation pathway and ultimately shape platelet adhesive behaviour and thrombotic outcomes.

## Overview of computational methods

### Model construction

Ligand-bound cryo-EM structures of full-length human α_IIb_β_3_ in bent-closed and open-extended conformations were used [37]. Missing residues were reconstructed using homology modeling [38, 39], and ligand-free systems were generated by removing the bound RGD motif from the ligand binding site. In total, four systems were prepared, representing bent and open conformations both with and without RGD. All models were solvated with the CHARMM-modified TIP3P water model and neutralized with 150 mM NaCl to approximate physiological ionic strength.

### Energy minimization and equilibration

Energy minimization was performed in GROMACS 2022.3 using the steepest descent algorithm [40]. Each system underwent a multistep equilibration protocol: an initial 250 ps NVT phase (at constant number of particles, N, constant volume, V, and temperature, T) at 310 K with gradually reduced positional restraints, followed by 175 ns of NPT equilibration (at constant number of particles, N, constant pressure, P, and temperature, T).

### Production simulations and structural analysis

To investigate the structural dynamics of α_IIb_β_3_, equilibrium molecular dynamics (MD) and steered molecular dynamics (SMD) simulations were run. Production simulations were carried out for 100 ns using a 2-fs integration time step. In SMD simulations, a constant pulling force of 83 pN was applied to the center of mass of the ligand-binding site, while the center of mass of the transmembrane helices was restrained.

Four simulation conditions were examined. In the *Control* condition, ligand-free α_IIb_β_3_ was simulated under equilibrium conditions without applied force, representing the basal, low-affinity conformation of integrin in circulating, inactive platelets. In the *Force* condition, ligand-free α_IIb_β_3_ was subjected to force, modeling force-induced integrin activation independent of ligand engagement (Fig. 1A, top). In the *RGD* condition, α_IIb_β_3_ was bound to an RGD motif under equilibrium conditions, representing integrin engagement with fibrinogen or fibrin clots in the absence of shear or contractility (Fig. 1A, bottom). In the *Force/RGD* condition, α_IIb_β_3_ was bound to an RGD motif and subjected to force, modeling integrin activation under shear or contractile force while engaged by ligands (Fig. 1C). For each condition, three independent simulation replicas were performed. Structural dynamics were quantified by calculating Cα root-mean-square deviation (RMSD) and root-mean-square fluctuation (RMSF), monitoring headpiece extension, and using principal component analysis (PCA). Dynamic cross-correlation matrices (DCCMs) were computed, and betweenness centrality of the residues was evaluated by constructing their interaction networks [41].

### Convergence assessment

Cα RMSD and RMSF were analyzed across three replicas per condition. Bootstrapping (1000 resamples per replica) was used to calculate 95% confidence intervals for RMSD values within and across replicas. Block analysis was performed by dividing each trajectory into consecutive time blocks, up to 25 ns each. Convergence of RMSF profiles was assessed by comparing residue fluctuations across sequential 10 ns windows.

## Results

### Effects of force and RGD on residue motional correlations in α_IIb_β_3_

**Fig. 2:**
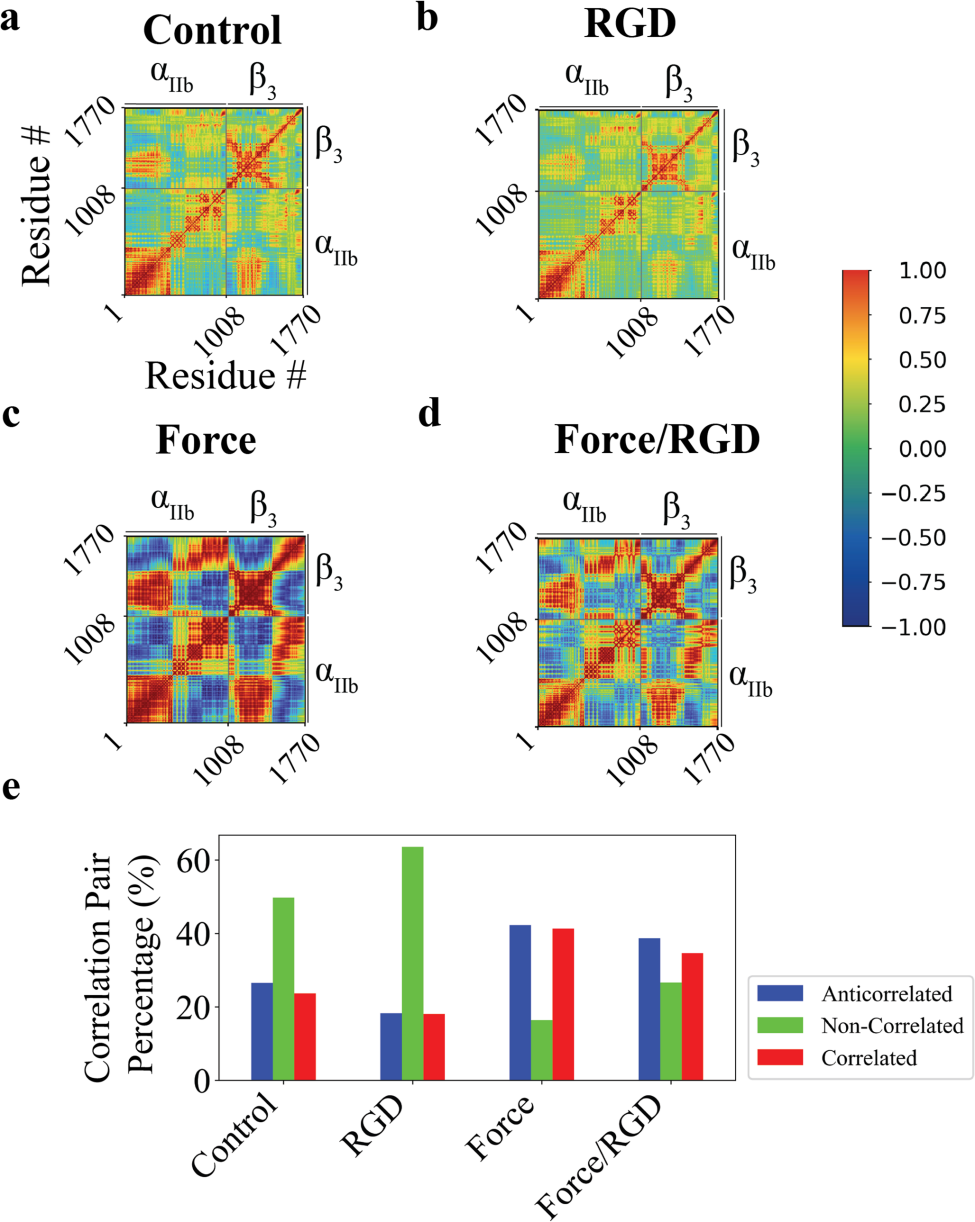
Normalized Dynamic Cross-Correlation (nDCC) maps of α_IIb_β_3_. (A-D) nDCC maps for the bent α_IIb_β_3_ integrin under four conditions: (**A**) control, (**B**) RGD-bound, (**C**) under applied force, and (**D**) combined Force/RGD condition. The maps are computed from three independent replicas per condition, using 1000 frames spanning the 0–100 ns simulation window. (**E**) Percentage of residue pair correlations in the bent conformation, divided into three groups based on nDCC values: red (0.25 < nDCC < 1), green ( −0.25 < nDCC < 0.25), and blue (−1 < nDCC < − 0.25). Data were calculated from the nDCC maps shown in panels A-D

To investigate the structural dynamics of α_IIb_β_3_ in response to force and/or ligand binding, we analyzed the normalized Dynamic Cross Correlation (nDCC) maps from all-atom MD simulations. The nDCC quantifies residue motion correlations, with values ranging from − 1 (strong anti-correlation) to 1 (strong correlation), and 0 indicating no correlation. In *Control*, ~ 50% of residue pairs showed non-correlated motions (Fig. 2A). RGD binding increased this to > 63%, while reducing strongly correlated pairs from ~ 50% to ~ 37% (Fig. 2B). By contrast, *Force* decreased non-correlated motions to ~ 16%, increasing correlated pairs to ~ 84% (Fig. 2C). In the combined *Force/RGD* condition, non-correlated pairs rose ~ 10% compared to *Force*, with a decrease in correlated pairs (Fig. 2D). (Fig. 2D). In summary, ligand binding increases non-correlated residue motions independent of force, but force application favors correlated motions (Fig. 2E). These findings suggest that α_IIb_β_3_ activation may follow distinct pathways depending on whether the stimulus is mechanical or biochemical.

## Force, not RGD, governs α_IIb_β_3_ extension and conformational stabilization

**Fig. 3:**
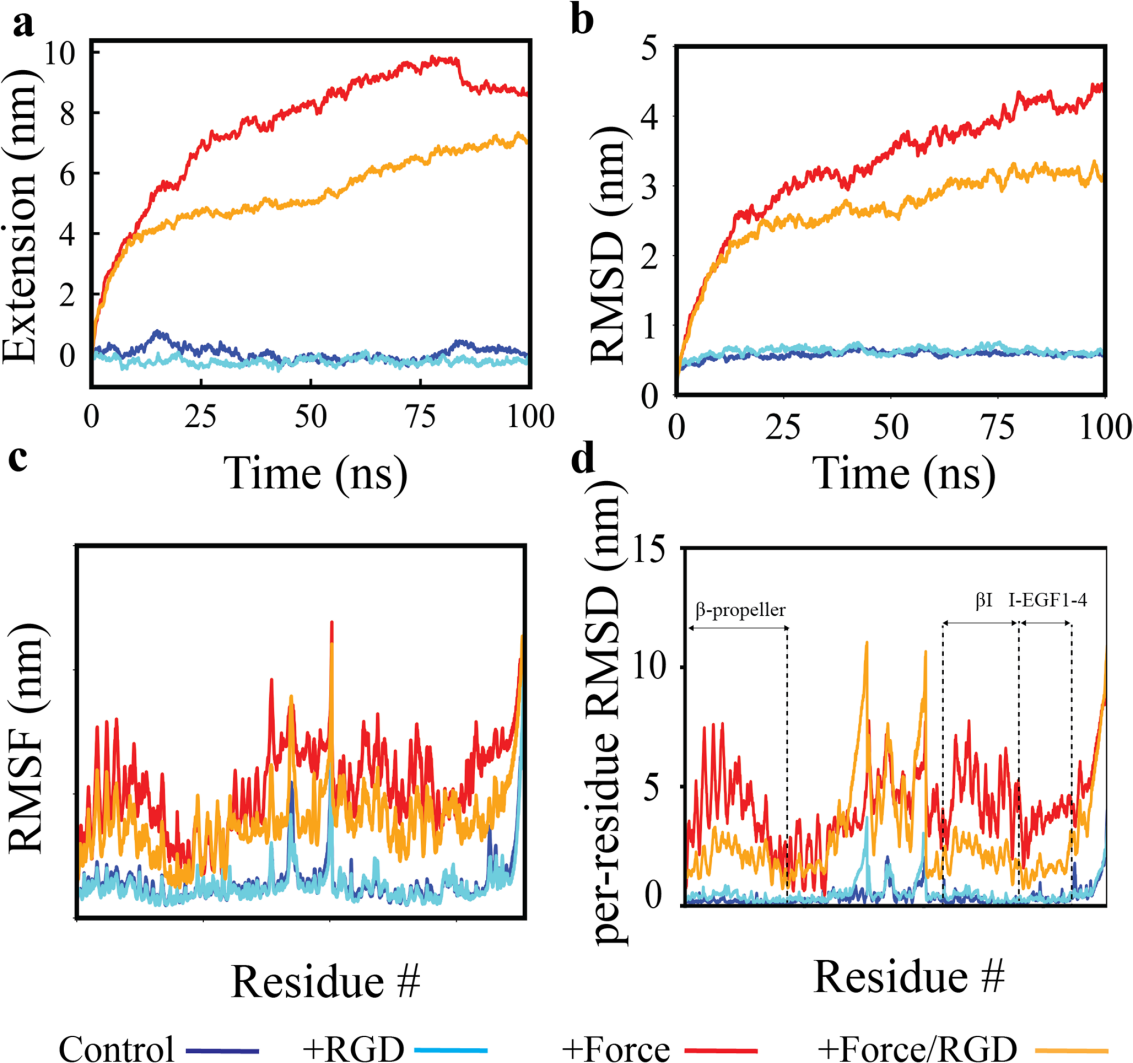
Effects of force and RGD binding on the structural dynamics of α_IIb_β_3_. Figure 3. (**A**) Extension vs. time under *Control*, *RGD*, *Force*, and *Force/RGD* conditions. Plotted are averages; maximum standard deviations (SDs) were 0.908 nm (*Control*), 1.446068 nm (*Force*), 0.871 nm (*RGD*), and 2.009 nm (*Force/RGD*). (**B**) Average Cα RMSD with respect to the input conformation. Maximum SDs were 0.134 nm (*Control*), 0.561 nm (*Force*), 0.192 nm (*RGD*), and 0.657 nm (*Force/RGD*). (**C**) Average Cα RMSF. Maximum SDs were 0.553 nm (*Control*), 0.375 nm (*Force*), 0.445 nm (*RGD*), and 0.491 nm (*Force/RGD*); minimum SDs were 0.0003 nm (*Control*), 0.0006 nm (*Force*), 0.0001 nm (*RGD*), and 0.0004 nm (*Force/RGD*) (**D**) Per-residue RMSD between input and final conformations. Maximum SDs were 1.672 nm (*Control*), 1.791 nm (*Force*), 1.537 nm (*RGD*), and 5.412 nm (*Force/RGD*); minimum SDs were 0.002 nm (*Control*), 0.010 nm (*Force*), 0.003 nm (*RGD*), and 0.043 nm (*Force/RGD*). All data were extracted from three replicas

Given that both mechanical force and ligand binding alter the structural dynamics of α_IIb_β_3_ (Fig. 2), we examined their impact on extension, along with per-residue displacements and fluctuations. By 100 ns, α_IIb_β_3_ extended to ~ 8 nm under Force and ~ 6.2 nm under Force/RGD, while *Control* and *RGD* showed negligible extension (Fig. 3A). Average Cα RMSD reached ~ 4.3 nm (*Force*) and ~ 3 nm (*Force/RGD*), compared with ~ 0.5 nm in *Control* and RGD (Fig. 3B; Figure S1). RMSD stability was confirmed via block averaging (Figure S2), SEM plateauing (Figure S3), and bootstrap resampling (Figures S4, S5). Differences in Cα RMSF across time intervals decreased (Figure S6). Average RMSF values mirrored extension, with *Force* (~ 1.08 nm) and *Force/RGD* (~ 0.80 nm) exceeding *Control* (~ 0.31 nm) and *RGD* (~ 0.29 nm) (Fig. 3C). Per-residue RMSD showed that the β-propeller and β-I headpiece, as well as I-EGF1-4, moved > 4 nm under *Force*; *Force/RGD* caused slightly smaller but notable displacements (Fig. 3D). *Control* and *RGD* displacements remained < 3 nm. *Force* applied to open α_IIb_β_3_ often reduced per-residue RMSD relative to *Control* (Figure S7), opposite to its effect on bent α_IIb_β_3_, indicating force-dependent stabilization of the open conformation [9, 42–52]. Together, these results indicate that force drives α_IIb_β_3_ extension by enhancing motions of key residues facilitating large-scale conformational changes. In open α_IIb_β_3_, force stabilizes the extended state in a strain-stiffening-like manner (Figure S7). *RGD* alone has minimal effect within 100 ns, while *Force/RGD* reduces motion relative to *Force* alone but remains above *Control* or *RGD*, highlighting force as the dominant cue for α_IIb_β_3_ extension when both stimuli are present.

### Principal component analysis reveals force-driven motions in α_IIb_β_3_

**Fig. 4:**
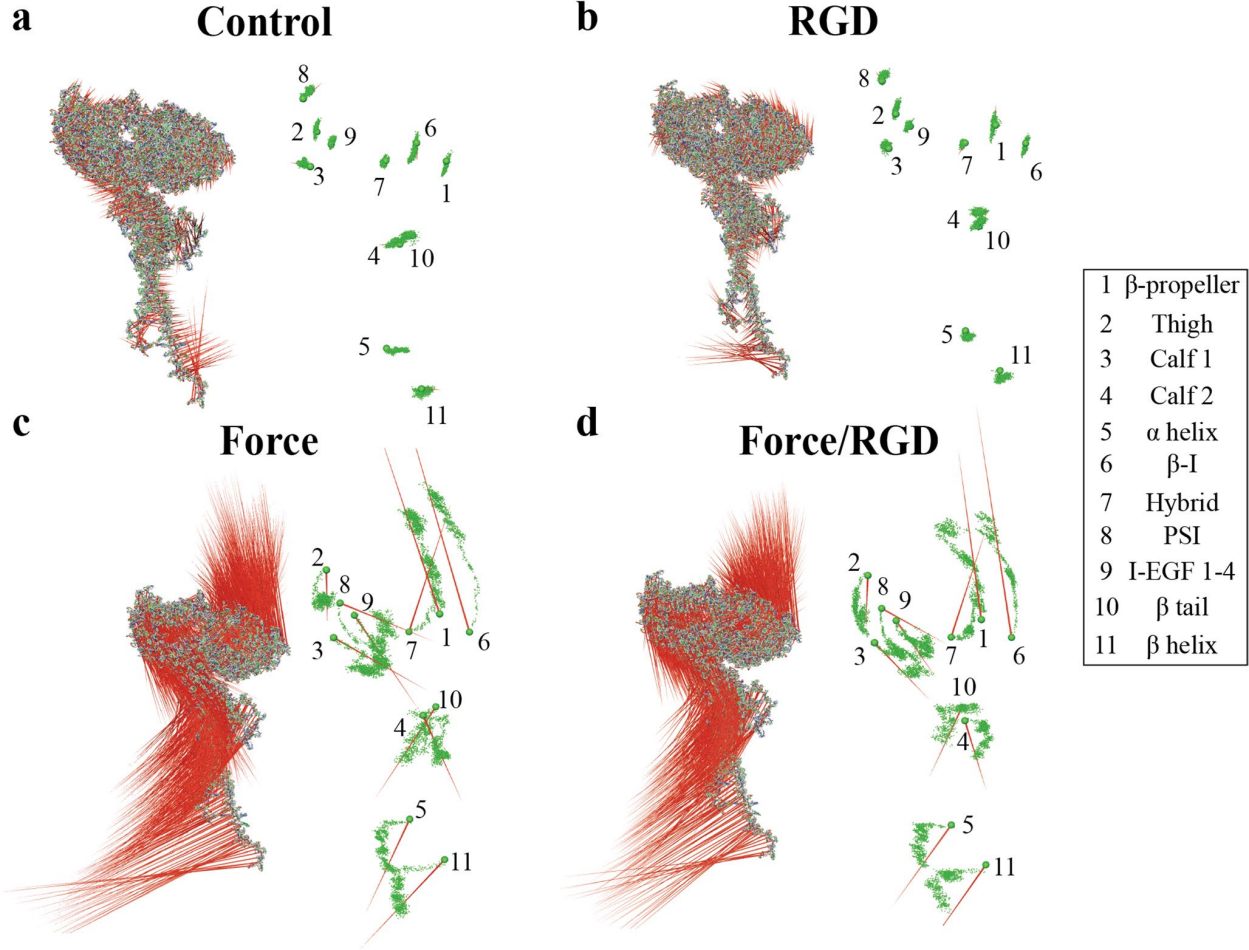
Porcupine plots of dominant motions from PCA. (**A**-**D**) The first eigenvector of bent conformation under (**A**) *Control*, (**B**) *RGD*, (**C**) *Force*, and (**D**) *Force/RGD*, alongside the center of geometry movement of each domain. Data were extracted from the 100 ns Cα trajectories of three independent equilibrium and steered MD simulations

To investigate the α_IIb_β_3_ conformational dynamics, we performed principal component analysis (PCA) on Cα atoms and individual α_IIb_β_3_ domains (Fig. 4). Arrows in the visualizations indicate motion direction and magnitude. In *Control*, bent α_IIb_β_3_ displayed only minor, non-directional movements, consistent with equilibrium fluctuations (Fig. 4A). *RGD* binding enhanced residue motions, particularly at the ligand-binding site (β-propeller and β-I) and the β_3_ transmembrane helix (Fig. 4B). *Force* further amplified these motions, with longer displacement vectors (Fig. 4C). The β-propeller and β-I domains moved along the direction of applied force, whereas the Thigh, PSI, I-EGF1-4, and Calf-1 domains shifted oppositely (Fig. 4C). Under *Force/RGD*, vector magnitudes increased in the head and lower leg domains, but motion directions remained similar to *Force* (Fig. 4D). Overall, PCA reveals that while both RGD and force enhance residue motions, mechanical force is the dominant driver of α_IIb_β_3_ conformational dynamics.

####  Force and RGD differentially modulate communication and flexibility in α_IIb_β_3_

**Fig. 5:**
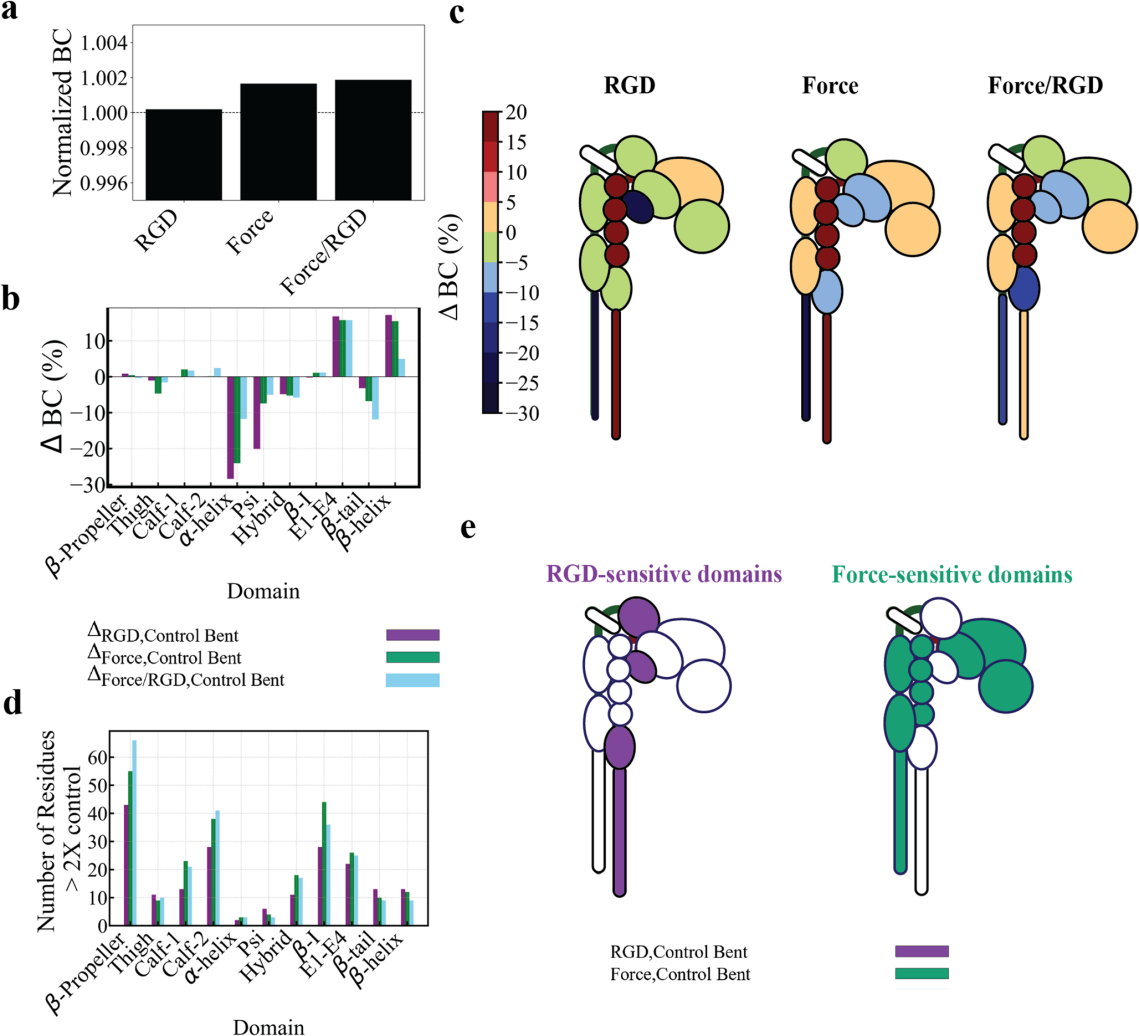
Betweenness centrality (BC) analysis. (**A**) Average BC values for bent integrin under different conditions, normalized by the control condition. (**B**) Percentage differences in average BC per domain compared to the control. (**C**) Cartoon representation of the percentage difference in average BC per domain compared to the control. Domains are colored according to the percentage change in betweenness centrality, with a color scale ranging from blue (−30.0%) indicating a significant decrease, through yellow (0%) for no change, to red (+ 20.0%) indicating a significant increase. (**D**) The number of residues in each domain with BC exceeding twice the control value. (**E**) Cartoon representation of integrin domains with color-coded BC showing force- and RGD-sensitive domains. Data were extracted from the Cα trajectories of three independent simulations

To examine how RGD binding and force influence the communication between α_IIb_β_3_ residues, we analyzed Betweenness Centrality (BC), which quantifies how often a residue lies on the shortest communication paths between others. High-BC residues act as communication hubs, whereas low-BC residues contribute less to internal signaling. Normalized average BC values (relative to *Control*) showed that RGD alone did not alter overall communication, while *Force* and *Force/RGD *enhanced it (Fig. 5A), consistent with increased residue motional correlations (Fig. 2). Domain-specific analysis revealed that *RGD*, *Force*, and *Force/RGD* decreased BC (decreased allosteric regulation) in the Thigh (~ 5%), α-helix (~ 11–28%), PSI (~ 6–20%), Hybrid (~ 5%), and β-tail (~ 12%) domains, while increasing BC (increasing allosteric regulation) in Calf-1 (~ 2%), β-I (~ 2%), EGF1–4 (~ 15%), and β-helix (~ 4–16%) domains (Fig. 5B, C). High-BC residues (≥ 2× Control mean) were most numerous under *Force* (243), followed by *Force/RGD* (240) and *RGD* (190), indicating that force enhances long-range structural communication and allosteric regulation. *Force* increased high-BC residues across multiple domains—β-propeller (55 vs. 43 in *RGD*), Calf-1 (23 vs. 13), Calf-2 (38 vs. 28), β-I (44 vs. 28), Hybrid (18 vs. 11), EGF1–4 (26 vs. 22), and α-helix (3 vs. 2)—consistent with increased rigidity in mechanically responsive regions (Fig. 5D, E). In contrast, *RGD* increased high-BC residues in only four domains (Thigh, PSI, β-tail, β-helix), suggesting selective reinforcement of local communication hubs in otherwise flexible regions. Compared with *Force* alone, *Force/RGD* further increased high-BC residues in β-propeller (66 vs. 55), Thigh (10 vs. 9), and Calf-2 (41 vs. 38) domains. These findings indicate that mechanical force predominantly strengthens global network connectivity and rigidity in key domains, while RGD fine-tunes communication within flexible regions. The additive effects observed under *Force/RGD*, particularly in β-propeller, Thigh, and Calf-2, highlight a cooperative role of mechanical and biochemical stimuli in coordinating allosteric regulation across α_IIb_β_3_.

#### Force and RGD stabilize the open conformation of α_IIb_β_3_

**Fig. 6:**
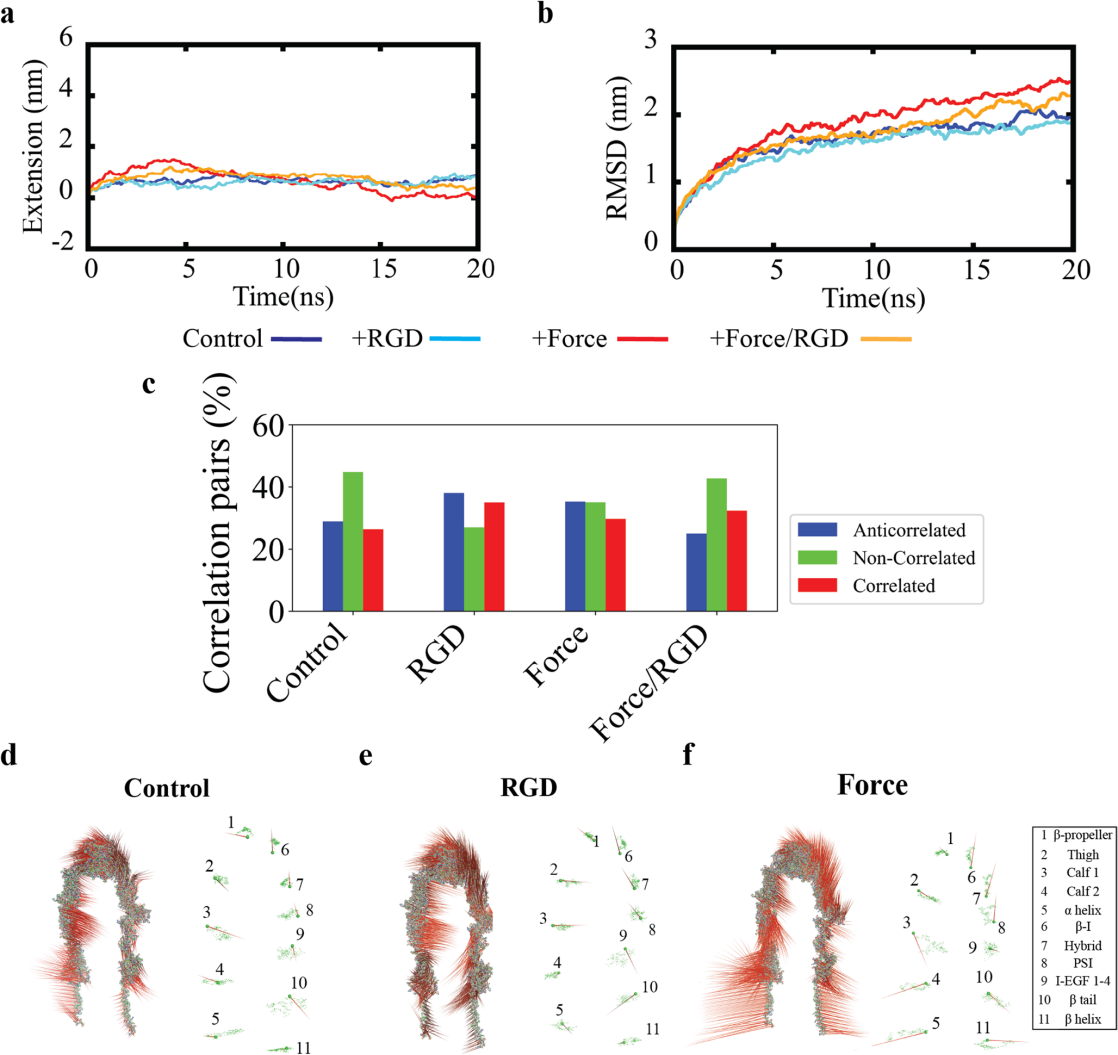
Effects of force and RGD binding on the open conformation of integrin. (**A**) Extension vs. time plot comparing *Control*, *RGD*, *Force*, and *Force/RGD*. (**B**) RMSD of Cα atoms under different conditions. (**C**) Percentages of correlated residue pairs in open conformation

To evaluate how force and RGD binding affects the open extended conformation of α_IIB_β_3_, we analyzed structural extension, Cα RMSD, and residue motions. Application of force caused a transient extension (~ 1.8 nm) but no sustained elongation beyond the fully extended state (Fig. 6A). Cα RMSD increased modestly under force (~ 2.5 nm vs. ~2.0 nm in Control), less pronounced than in bent α_IIb_β_3_ (Fig. 3B). Ligand binding reduced RMSD in both *Force/RGD* and *RGD*-only conditions (~ 1.8 nm), consistent with ligand-mediated stabilization [46, 47, 49–52]. *RGD* also decreased non-correlated residue motions in the open state (~ 43% to ~ 22%) (Fig. 6C, S8), opposite to its effect in bent α_IIB_β_3_ (Fig. 2E). *Force* alone slightly reduced non-correlated motions, and combining *Force/RGD* caused a minor increase (~ 41% vs. ~38% in *Force*). Compared with bent α_IIb_β_3_, the open *Control* displayed larger domain displacements (Fig. 6D vs. 4 C), reflecting lower stability. *RGD* promoted headpiece dynamics but dampened motions in Calf-2 and the α-helix (Fig. 6E), again opposite to its effect in the bent conformation. *Force* primarily increased motions in the lower legs and helices, though less than in the bent conformation (Fig. 6D, F vs. 4 A, C). Overall, force and ligand binding stabilize α_IIb_β_3_ in a conformation-dependent manner: in the bent state, force extends and stabilizes the integrin while RGD increases local flexibility; in the open state, both stimuli reinforce structural stability, with maximal effect under combined *Force/RGD* (Fig. 6D–F, S9).

## Discussion

Precise regulation of α_IIb_β_3_ activation is essential for balancing platelet adhesion and aggregation across diverse vascular environments, enabling rapid hemostasis while preventing pathological thrombosis. Although biochemical ligands and mechanical forces are both known to regulate integrin activation, how α_IIb_β_3_ distinguishes and integrates these cues at the structural level has remained unclear. By isolating ligand binding and force in all-atom simulations, our results demonstrate that α_IIb_β_3_ does not rely on a single, unified activation pathway. Instead, biochemical and mechanical stimuli act through distinct but complementary mechanisms that converge on stabilization of the extended, open conformation.

From our simulations, mechanical force emerged as the dominant driver of long-range allosteric reorganization. Force strongly increased correlated residue motions across the ectodomain (Fig. 2), promoted head–leg separation and extension (Fig. 3), and reinforced long-range structural connectivity (Fig. 5). Network analysis further confirmed that force enhances allosteric regulation by increasing the number and distribution of communication hubs in several functional domains. Importantly, force played a dual role: while it destabilized the bent conformation to facilitate extension, it stabilized the open conformation by reducing residue-level fluctuations (Fig. 6). This strain-stiffening–like behavior is consistent with experimental observations showing that shear flow and platelet-generated contractile forces promote integrin extension and stabilize ligand-bound states under load [53, 54]. These findings provide a molecular framework for how shear forces in the vasculature—ranging from venous to pathological arterial flows—can directly activate or stabilize α_IIb_β_3_ by reorganizing its internal dynamics, enabling the receptor to sustain load while transmitting mechanical signals across distant domains.

In contrast, RGD binding did not promote global coordination or extension on the simulated timescale, but instead increased non-correlated residue motions (Fig. 2) and selectively reinforced local communication hubs within flexible regions, particularly within the Thigh, PSI, and β-tail domains (Figs. 5). The involvement of the β-tail domain is notable, as mutations in this region are known to modulate physiological ligand binding and signaling [55]. This behavior suggests that ligand engagement alone softens the ectodomain, increasing conformational plasticity rather than enforcing rigidity. Such flexibility may allow α_IIb_β_3_ to sample activation-competent states or accommodate force-bearing ligands, consistent with experimental observations that resting platelets can adhere to immobilized fibrinogen or fibrin under load. Importantly, when combined with force, RGD fine-tuned force-induced communication and dynamics, suggesting a cooperative interaction in which biochemical cues modulate, rather than replace, mechanically driven activation.

Finally, our analysis of the open conformation revealed that the roles of force and ligand binding are strongly state-dependent. While force destabilized the bent state to drive extension, it stabilized the open state by suppressing excessive motions (Fig. 6), consistent with strain-stiffening behavior. RGD binding further stabilized the open conformation by reducing uncorrelated residue motions (Fig. 6). Together, these results support a model in which α_IIb_β_3_ integrates biochemical and mechanical signals through distinct but convergent mechanisms. Such interplay is highly relevant physiologically, as platelet α_IIb_β_3_ must discriminate between soluble fibrinogen, which does not trigger activation, and immobilized ligands or force-bearing adhesions that promote firm attachment and signaling [56–62]. Overall, our findings are consistent with prior experimental and computational studies demonstrating force-driven long-range allostery [9, 42, 43, 52, 63] and ligand-induced dynamic priming [45, 64], and they provide an integrated mechanistic framework for how α_IIb_β_3_ coordinates mechanical and biochemical inputs to regulate platelet function [50, 52, 65]. In the complex mechanical environment of the vasculature, the balance between force-induced global coordination and ligand-induced local flexibility may determine how platelets achieve rapid yet tightly regulated adhesion, with important implications for both hemostasis and thrombosis.

### Limitations of the study

This study relies on all-atom molecular dynamics simulations conducted on microsecond-subscale trajectories, which, while sufficient to capture early activation events, may not fully resolve slower conformational transitions of α_IIb_β_3_ that occur on longer timescales. In particular, ligand-driven activation pathways may be underestimated within the simulated windows. Extending simulations to 200 ns and applying lower forces (20 pN) preserved key differences in residue correlations between force and no-force conditions but did not reproduce full headpiece extension or sustained stabilization of the open conformation (Figure S10). Enhanced sampling approaches, such as umbrella sampling or metadynamics, will likely be required to explore these slower transitions.

Mechanical force in our simulations was applied in a simplified and idealized manner, along a fixed direction and at constant magnitude. In vivo, α_IIb_β_3_ can experience heterogeneous force profiles arising from shear flow, ligand anchoring, cytoskeletal tension, and membane. Exploring alternative loading geometries could help clarify how integrin activation depends on heterogeneous forces. Additionally, prior studies indicate that integrins often relax toward the bent conformation once force is released, but whether a threshold force exists that drives irreversible activation remains unresolved and may be critical for distinguishing reversible from irreversible platelet activation. Exploring variations in force magnitude and relaxation dynamics could clarify these behaviors.

Key cellular components known to regulate integrin activation were not explicitly included. Cytoskeletal interactions essential for inside-out signaling were omitted, as the present work focuses on ligand-induced activation and outside-in signaling. The models also lack an explicit lipid bilayer; however, prior studies and our supplementary simulations (Figure S11) suggest that force-induced extension and residue-level dynamics are largely conserved in membrane contexts. In contrast, RGD binding and combined force/RGD conditions may be more sensitive to membrane effects [66]. 

Finally, while network and correlation analyses provide insights into allosteric regulation, they infer functional coupling from dynamical correlations rather than directly measuring signaling outcomes. Experimental validation using force-clamp assays, mutagenesis of predicted communication hubs, or integrin activation reporters will be essential to confirm the mechanistic roles proposed here and to establish their physiological relevance. Future studies employing longer simulations, enhanced sampling, explicit membrane and cytoskeletal models, and experiments will be necessary in the future.

## Materials and methods

### Construction of the integrin systems

Simulations were performed for bent and open integrins with and without RGD at the ligand-binding site. The input structures for the four α_IIb_β_3_ conditions are deposited as PDB files in our GitHub repository (see Data Availability). For the bent conformations, the simulation box dimensions ranged from 15.7 to 15.9 nm in X, 13.0 to 13.9 nm in Y, and 32.6 to 32.9 nm in Z. For the open conformations, box dimensions ranged from 14.5 to 15.0 nm in X, 15.0 to 15.8 nm in Y, and 425.8 to 429.1 nm in Z. For simulations under force, the vertical dimension was extended by 10 nm. To ensure the force was applied parallel to the transmembrane α-helix, all conformations were aligned such that the helix was oriented along the Z-axis, and a constant force was applied along this axis. In these simulations, applying force along the Z axis mimicked the physiological mechanical extension experienced by integrins under shear stress in platelets. This orientation facilitates the transition of α_IIb_β_3_ from the bent to the extended conformation and is consistent with previous computational and biophysical studies—including SMD, atomic force microscopy, and biomembrane force probe experiments [67–69]—that applied forces along the head-to-tail axis of the protein in the pico-Newton range to investigate integrin extension.

The bent system without RGD contained 680,864 atoms (26,834 protein and 654,030 solvent and ions), whereas the bent system with RGD contained 670,952 atoms (26,944 protein and 644,008 solvent and ions). The open system without RGD contained 953,911 atoms (26,577 protein and 927,334 solvent and ions), and the open system with RGD contained 934,622 atoms (26,688 protein and 907,934 solvent and ions).

### Energy minimization

Energy minimization was performed to relax the systems and eliminate steric clashes prior to molecular dynamics simulations. The steepest descent algorithm was used [40], proceeding until the maximum force on any atom fell below 1000 kJ mol⁻¹ nm⁻¹ or 50,000 steps were reached, with a step size of 0.002 ps. Non-bonded interactions were computed using the Verlet cutoff scheme [70, 71], with van der Waals interactions treated using a force-switching function from 1.0 to 1.2 nm and electrostatics calculated via the Particle Mesh Ewald method [72]. The neighbor list was updated every 10 steps with a 1.2 nm cutoff. Hydrogen bonds involving covalent hydrogens were constrained using the LINCS algorithm [73], and harmonic positional restraints with a force constant of 1000 kJ mol⁻¹ nm⁻² were applied to the protein backbone heavy atoms.

### Equilibration

Following energy minimization, the systems underwent a multistep equilibration procedure designed to gradually stabilize temperature, pressure, and density while minimizing structural perturbations before production simulations. The equilibration was conducted in two phases: an initial phase under the canonical (NVT) ensemble, followed by an extended phase under the isothermal-isobaric (NPT) ensemble. This stepwise approach was chosen based on standard CHARMM-GUI protocols [74] and previous studies [38, 39, 69], which showed that gradually releasing positional restraints and progressively increasing time steps improve stability and prevent abrupt conformational distortions.

In the NVT phase (Steps 1–2), initial velocities were generated at 310 K with a random seed to ensure a Boltzmann distribution. In Step 1, 4000 kJ mol⁻¹ nm⁻² position restraints were applied to protein heavy atoms. The system was integrated over 125,000 steps with a 0.001 ps time step (125 ps), maintaining temperature with the Berendsen thermostat (coupling time τ = 1.0 ps) [75]. In Step 2, the restraints were reduced to 2000 kJ mol⁻¹ nm⁻², and the simulation continued for 125 ps under identical thermostat conditions, without regenerating velocities.

The NPT phase (Steps 3–6) adjusted pressure and density. Step 3 applied restraints of 1000 kJ mol⁻¹ nm⁻² over 125 ps (0.001 ps timestep), using isotropic pressure coupling with the Berendsen barostat (target 1.0 bar, τ = 5.0 ps, compressibility 4.5 × 10⁻⁵ bar⁻¹). Steps 4–6 gradually reduced restraints in three stages (500 → 200 → 50 kJ mol⁻¹ nm⁻²), each integrated for 250,000 steps (0.002 ps timestep, 500 ps per step). Temperature and pressure coupling remained constant throughout. 

Throughout all equilibration steps, non-bonded interactions were calculated using the Verlet cutoff scheme [70], van der Waals interactions incorporated a force-switching function from 1.0 to 1.2 nm, and electrostatics were treated using the Particle Mesh Ewald method with a 1.2 nm real-space cutoff [72]. Neighbor lists were updated every 20 steps with a 1.2 nm cutoff. Hydrogen bonds were constrained using LINCS [73], and positional restraints were applied to prevent excessive conformational drift. The total equilibration time was 2.0 ns (250 ps NVT + 1.75 ns NPT).

### Production simulations

Production simulations were conducted using both constant-force steered molecular dynamics (SMD) and equilibrium molecular dynamics (MD). The simulations were run for 100 ns, employing a time step of 0.002 ps across 50,000,000 steps. This 100 ns duration, while insufficient to capture long-term conformational transitions, provided adequate sampling of short-timescale structural dynamics.

In the SMD simulations, a constant pulling force of 50 kJ mol⁻¹ nm⁻¹ (approximately 83 pN) was applied to the center of mass of residues forming the ligand-binding site, along the Z axis. This pulling group included residues from the metal ion-dependent adhesion site (MIDAS: E220, S121, S123, D119, D251), the ligand-associated metal ion-binding site (LIMBS: D217, N215, D158, P219), and the adjacent to MIDAS site (ADMIDAS: D126, D127, M335). The center of mass of the transmembrane helices (residues 967–988 and 1704–1723), defined as the reference group, was restrained along the Z axis while remaining free to move in the X and Y axes, mimicking confinement by the plasma membrane. For simulations in the presence of the RGD motif (i.e., *RGD* and *Force/RGD* conditions), the peptide was positioned within the binding site, and the force was applied to both the center of mass of RGD and the pulling group (see Fig. 1A).

For equilibrium MD simulations, no external force was applied. Pressure coupling was omitted in both SMD and equilibrium MD, with the simulations conducted in the NVT ensemble. Similar to the equilibration steps discussed above, the LINCS algorithm was employed to constrain covalent bonds involving hydrogen [73], enabling a simulation timestep of 2 fs. Non-bonded interactions were treated with a Lennard-Jones potential with a cutoff at 1.2 nm and a force-switching transition from 1.0 nm to 1.2 nm, while short-range electrostatic interactions used a 1.2 nm cutoff. Long-range electrostatics were computed using the Particle Mesh Ewald method [72].

We utilized VMD, MDAnalysis, PyMol, GROMACS analysis tools, and custom scripts to analyze and visualize simulation trajectories for quantitative analysis [76–79].

### RMSD data processing and bootstrapping

Root-mean-square deviation (RMSD) data were analyzed using a bootstrap resampling approach in MATLAB (version R2023b).

 For each replica, 1000 bootstrap samples were randomly sampled with replacement from the original RMSD values. The mean of each bootstrap sample was calculated, yielding a bootstrap distribution of mean RMSD values (Figure S4). The 95% confidence interval (CI) for the mean RMSD was calculated from the 2.5th and 97.5th percentiles of the bootstrap distribution.

In addition, bootstrap resampling was carried out within each replica, and the resulting resampled values were then combined across all replicas into a single distribution (Figure S5). 

### RMSF difference analysis and convergence assessment

Root mean square fluctuations (RMSF) of Cα atoms were computed for each replica over four consecutive 10 ns time intervals (0–10, 10–20, 20–30, and 30–40 ns). To assess convergence, absolute differences in RMSF values between successive time intervals were calculated: 0–10 ns vs. 10–20 ns, and 20–30 ns vs. 30–40 ns. These differences were aggregated across all replicas and visualized using boxplots (Figure S6). A diminishing distribution of RMSF differences between earlier intervals (0-10ns vs. 10–20 ns) and later intervals (20–30 ns vs. 30–40 ns) of the boxplots indicates convergence of the system: smaller differences at later intervals suggest that residue fluctuations have stabilized over time, whereas larger differences imply incomplete sampling and ongoing structural rearrangements.

### Dynamic cross correlation analysis

Normalized Dynamic cross correlation (nDCC) maps were generated to analyze pairwise correlations between residues using the CorrelationPlus package [80]. The DCC between residue pairs was calculated as follows:

$${C_{ij}}=\frac{{\langle \Delta {{\mathbf{r}}_i} \cdot \Delta {{\mathbf{r}}_j}\rangle }}{{\sqrt {\langle \Delta {\mathbf{r}}_{i}^{2}\rangle \langle \Delta {\mathbf{r}}_{j}^{2}\rangle } }}$$

where Δr_i_ represents the displacement of residue *i* from its average position throughout the simulation. The nDCC was calculated from the average of three independent replicas. The resulting matrix consists of correlation coefficients ranging from − 1.0 to + 1.0, reflecting the extent of coupling between the positional fluctuations of residues *i* and *j*. A positive *C*_*ij*_ approaching + 1.0 indicates correlated motion, meaning the residues move together in the same direction or exhibit coordinated movement. Conversely, a negative *C*_*ij*_ approaching − 1.0 shows anti-correlated motion, where residues move in opposite directions or display inversely related movements. When *C*_*ij*_ falls within approximately − 0.25 to + 0.25, it suggests a minimal coupling between the motions of the residues.

#### Betweenness centrality

Betweenness centrality (*C*_*B*_) was calculated to identify key residues mediating protein structure communication [81]. In this graph-theoretical approach, *C*_*α*​_ atoms were treated as nodes, and edges were formed between them based on two criteria: (i) the dynamical correlation (∣*C*_*ij*_∣) exceeded a threshold (e.g., 0.30), and (ii) the spatial distance (*d*_*ij*_​) between residues was below a specified cutoff (e.g., 7 A˚). The correlation threshold (|C_*ij*_| > 0.30) ensures that only sufficiently strong dynamic correlations are considered, filtering out weak or random correlations that may not be functionally relevant. The spatial distance cutoff (d_*ij*_ < 7 Å) is selected based on the average distance within which noncovalent interactions, such as hydrogen bonding and van der Waals contacts, typically occur in protein structures. The edges were assigned weights as:

$${w_{ij}}= - \log (|{C_{ij}}|)$$

Betweenness centrality for a residue *v* was then computed as:

$${C_B}(v)=\sum\limits_{{s \ne v \ne t}} {\frac{{{\sigma _{st}}(v)}}{{{\sigma _{st}}}}}$$

where *σ*_*st*_ is the total number of shortest paths between residues *s* and *t*, and *σ*_*st*_ is the number of those paths that pass through *v*. The calculation was implemented using the NetworkX library [41]. 

## Supplementary Information

Below is the link to the electronic supplementary material.

Supplementary Material 1 (PNG261 KB)

Supplementary Material 2 (PNG1.50 MB)

Supplementary Material 3 (PNG397 KB)

Supplementary Material 4 (PNG140 KB)

Supplementary Material 5 (PNG1.00 MB)

Supplementary Material 6 (PNG125 KB)

Supplementary Material 7 (PNG125 KB)

Supplementary Material 8 (PNG181 KB)

Supplementary Material 9 (PNG401 KB)

Supplementary Material 10 (PNG238 KB)

Supplementary Material 11 (PNG224 KB)

Supplementary file 12 (DOCX 32.9 KB)

## Data Availability

Full-length all-atom structures of integrin α_IIb_β_3_, molecular dynamics simulation trajectories, and analysis scripts are available on GitHub [https://github.com/Reza9248/Alpha2bbeta3-integrin-conformations.git].
