# Supplementary figures and images for: All-atom simulations reveal distinct pathways for αIIbβ3 activation by biochemical vs. mechanical cues
Source: Cell Mol Life Sci. 2026 Mar 6;83(1):157. doi: 10.1007/s00018-026-06138-9 (PMC13003053; doi:10.1007/s00018-026-06138-9)

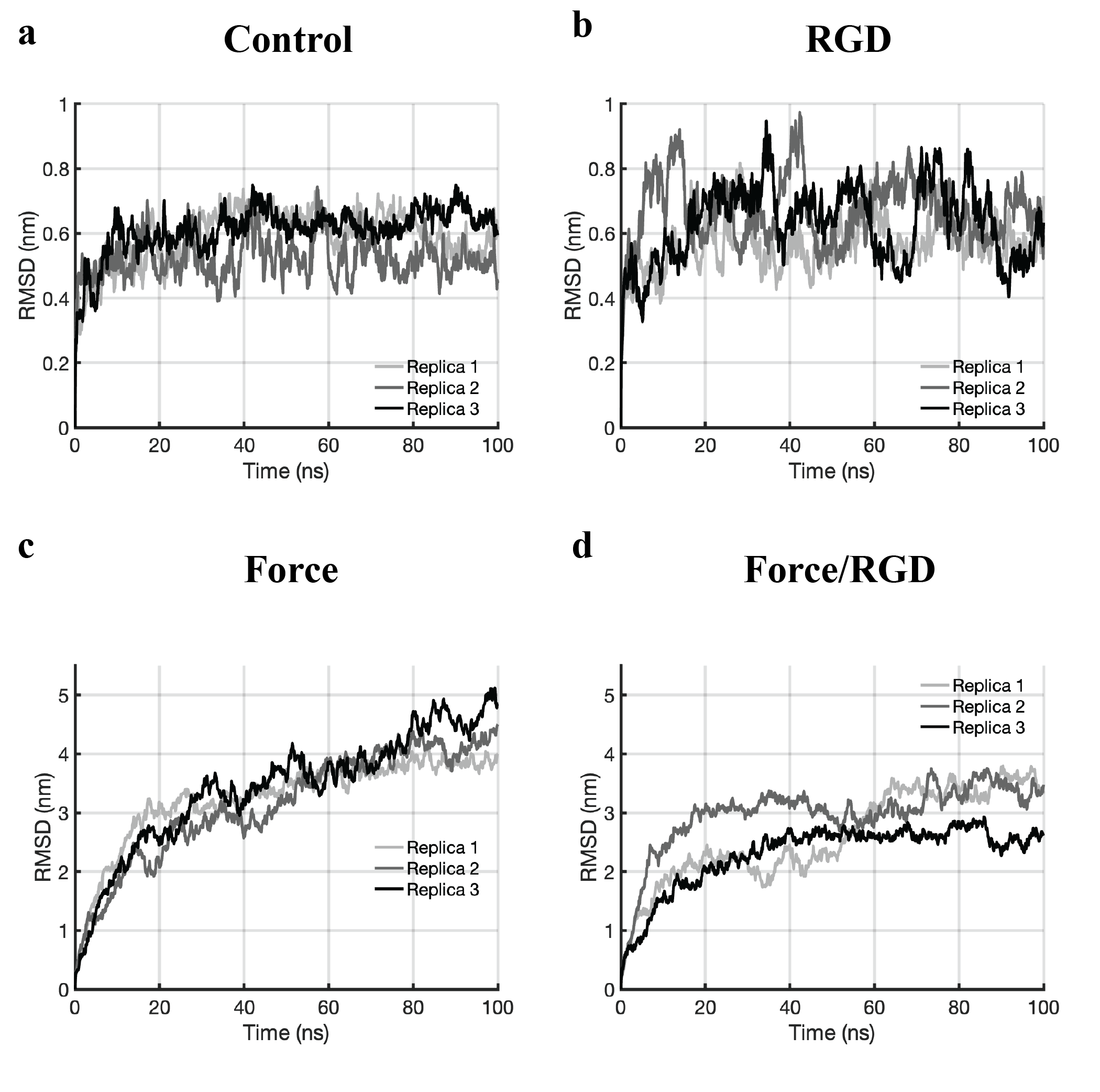

Supplement: Supplementary file 1 — Supplementary Material 1 (PNG261 KB) [file 18_2026_6138_MOESM1_ESM.png]

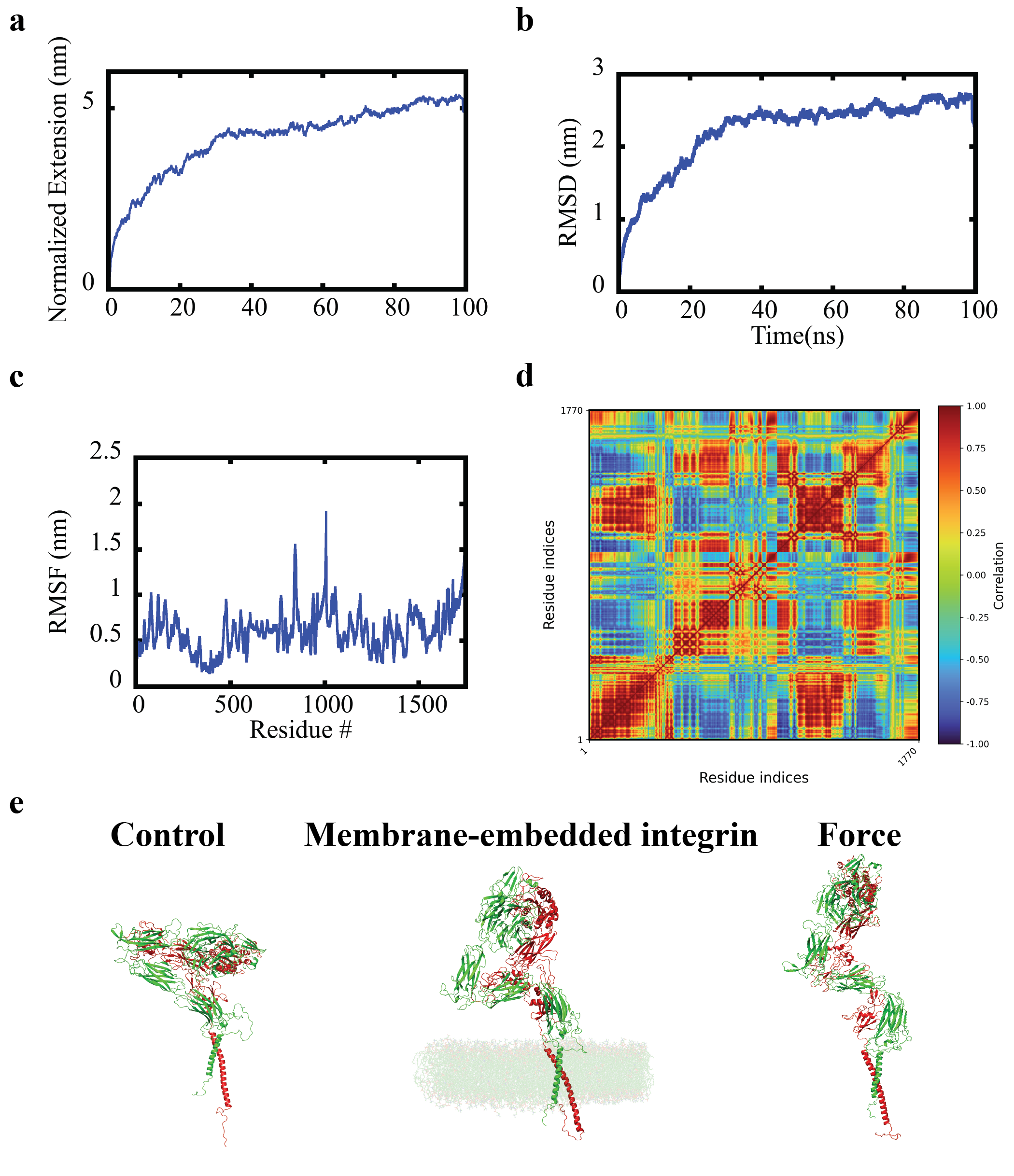

Supplement: Supplementary file 2 — Supplementary Material 2 (PNG1.50 MB) [file 18_2026_6138_MOESM2_ESM.png]

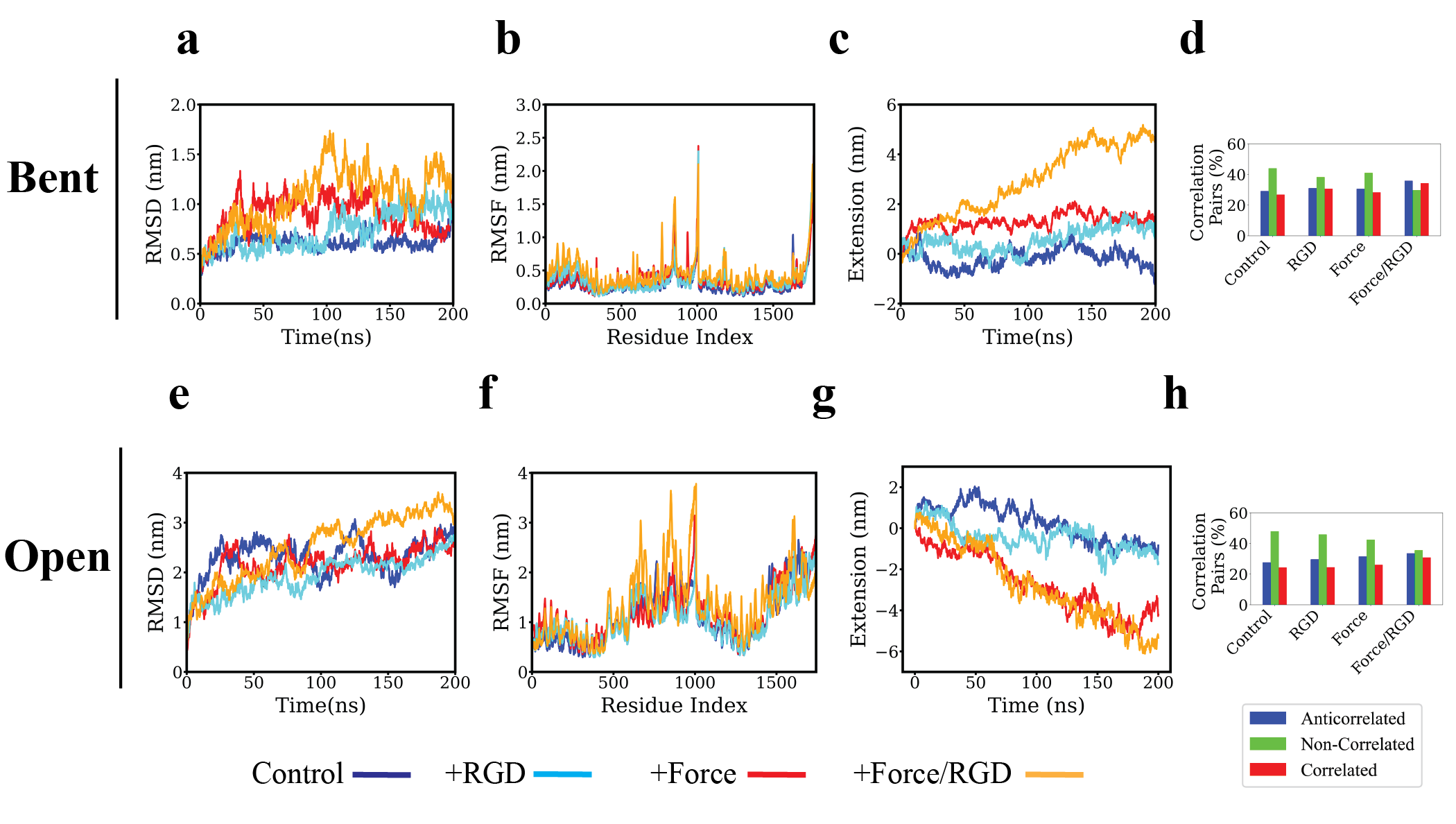

Supplement: Supplementary file 3 — Supplementary Material 3 (PNG397 KB) [file 18_2026_6138_MOESM3_ESM.png]

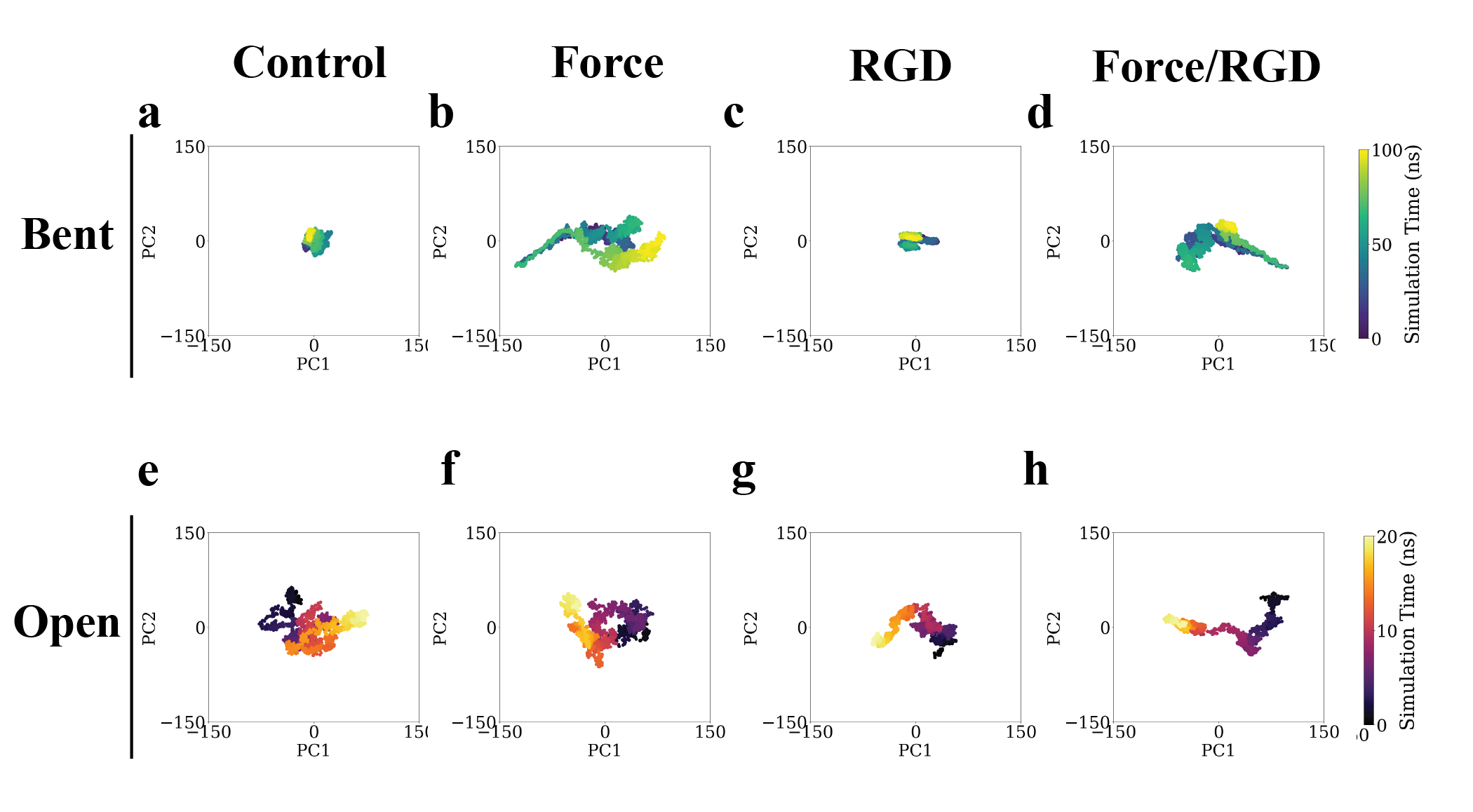

Supplement: Supplementary file 4 — Supplementary Material 4 (PNG140 KB) [file 18_2026_6138_MOESM4_ESM.png]

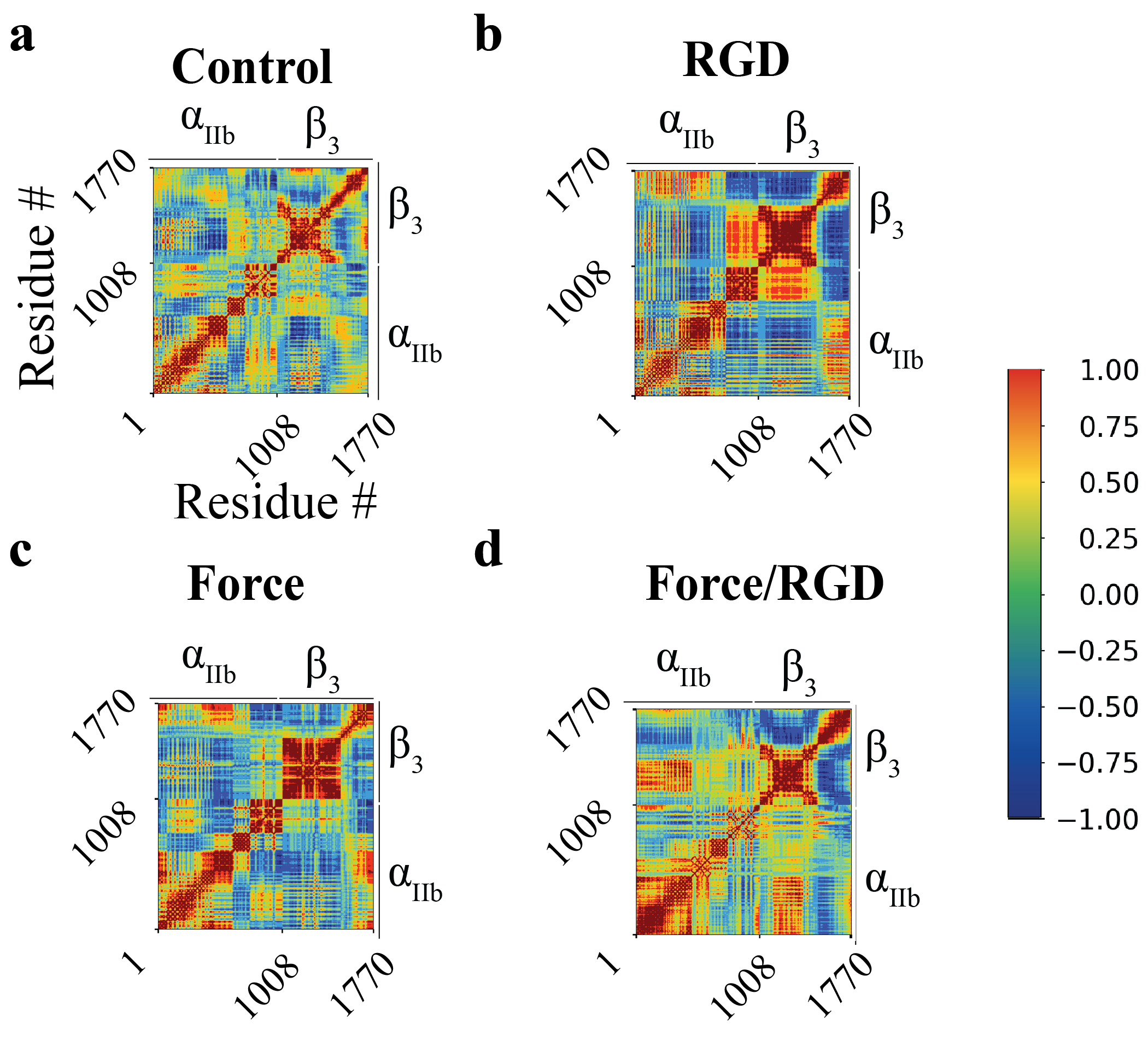

Supplement: Supplementary file 5 — Supplementary Material 5 (PNG1.00 MB) [file 18_2026_6138_MOESM5_ESM.png]

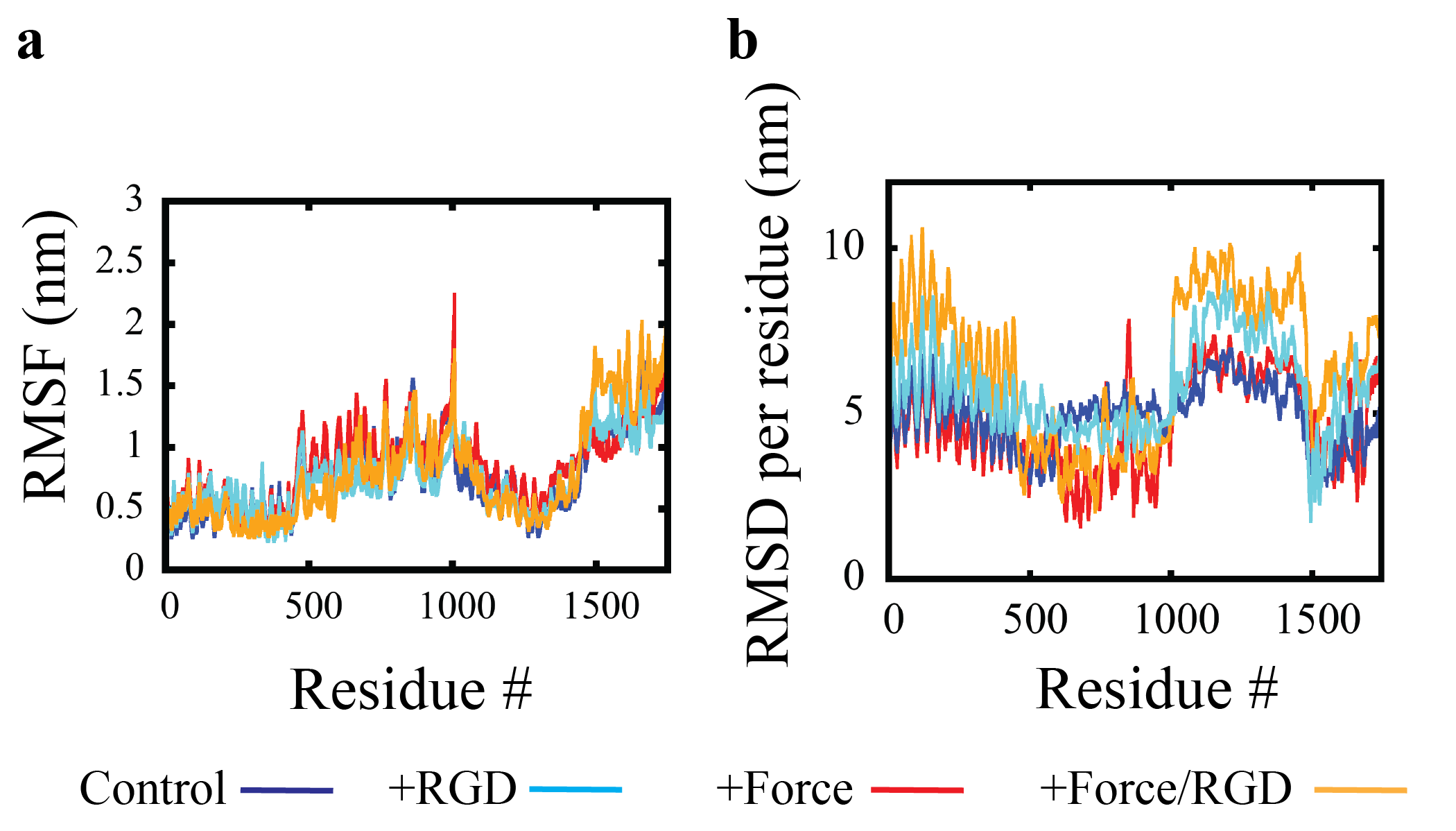

Supplement: Supplementary file 6 — Supplementary Material 6 (PNG125 KB) [file 18_2026_6138_MOESM6_ESM.png]

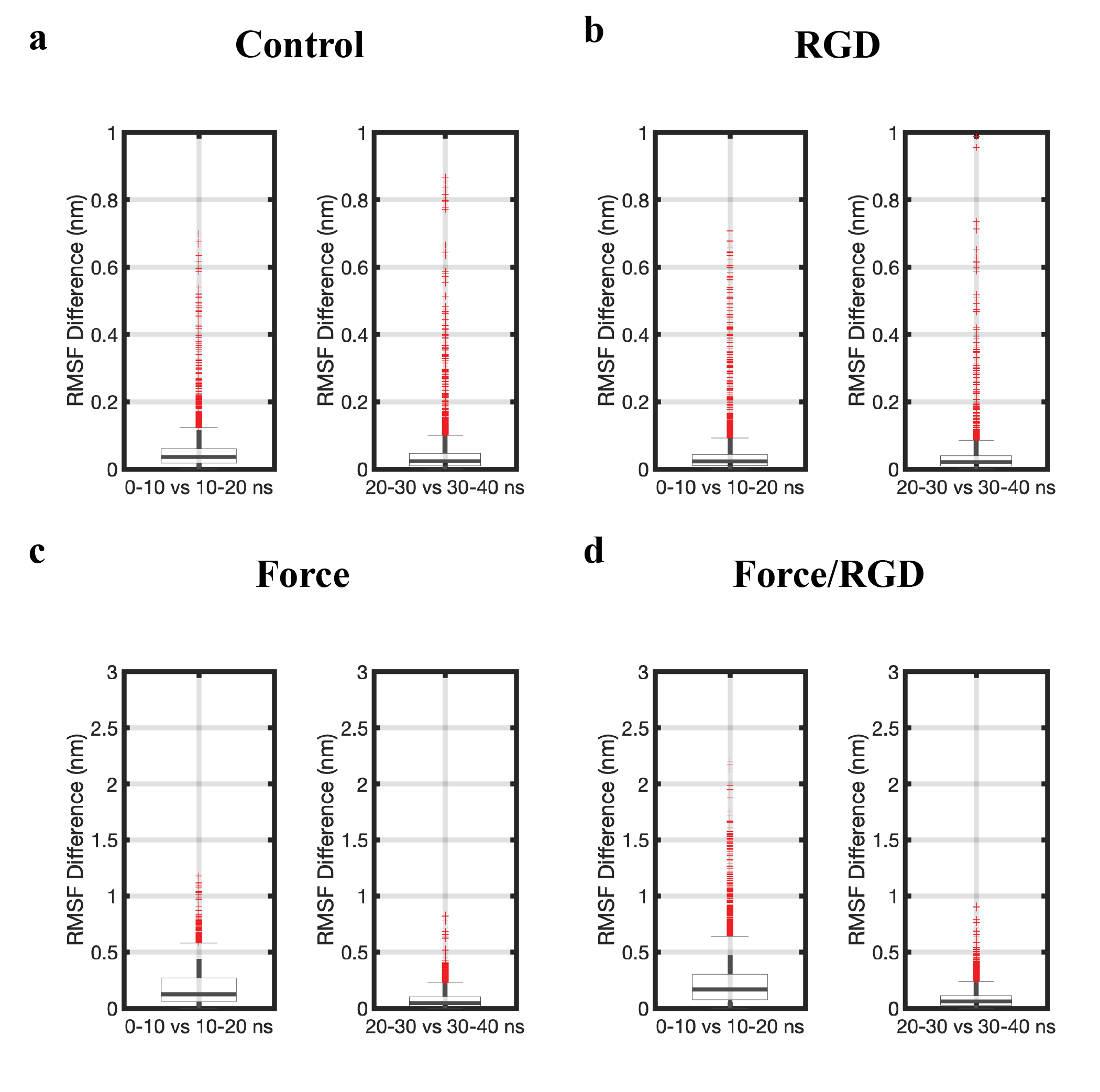

Supplement: Supplementary file 7 — Supplementary Material 7 (PNG125 KB) [file 18_2026_6138_MOESM7_ESM.png]

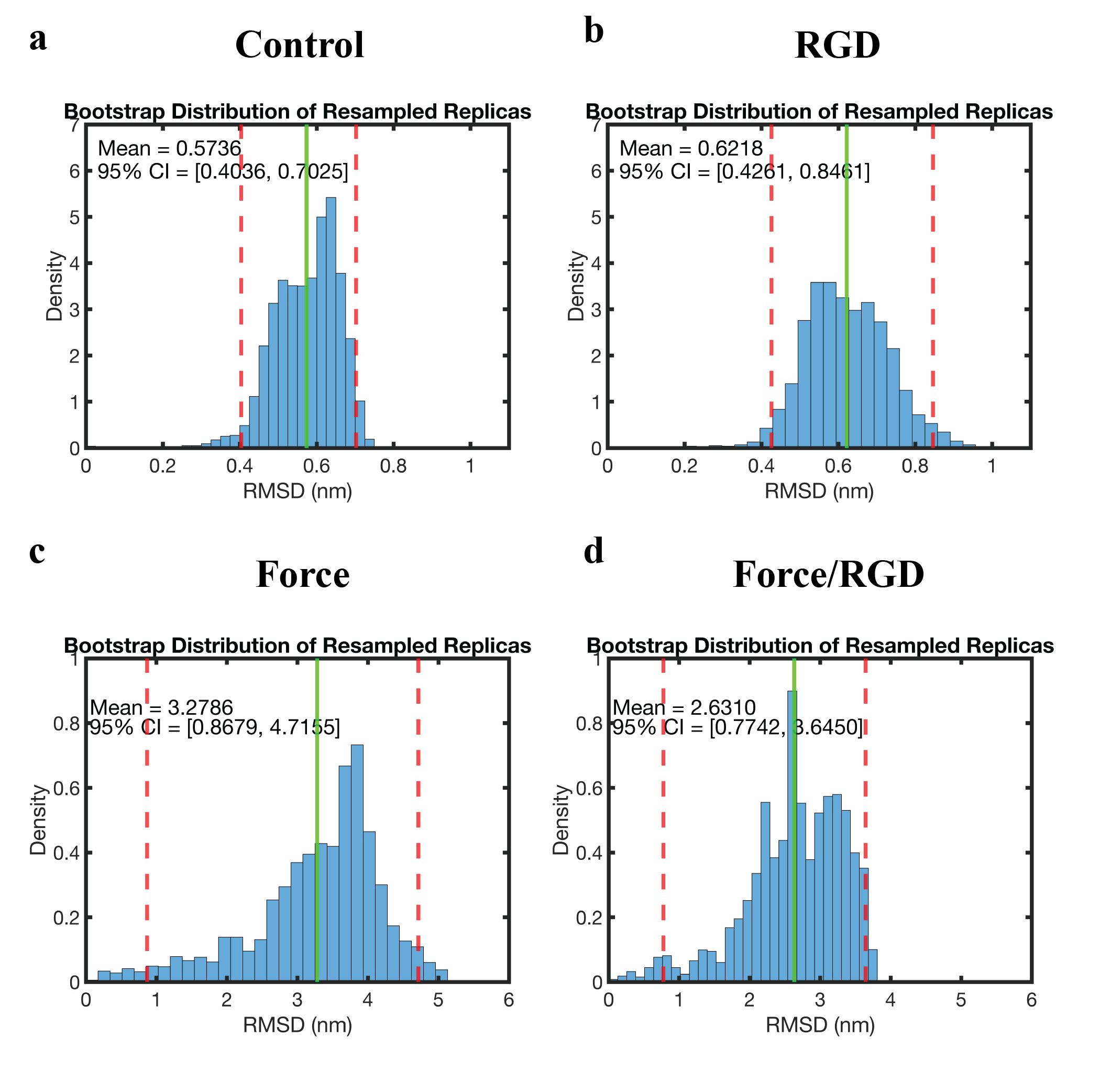

Supplement: Supplementary file 8 — Supplementary Material 8 (PNG181 KB) [file 18_2026_6138_MOESM8_ESM.png]

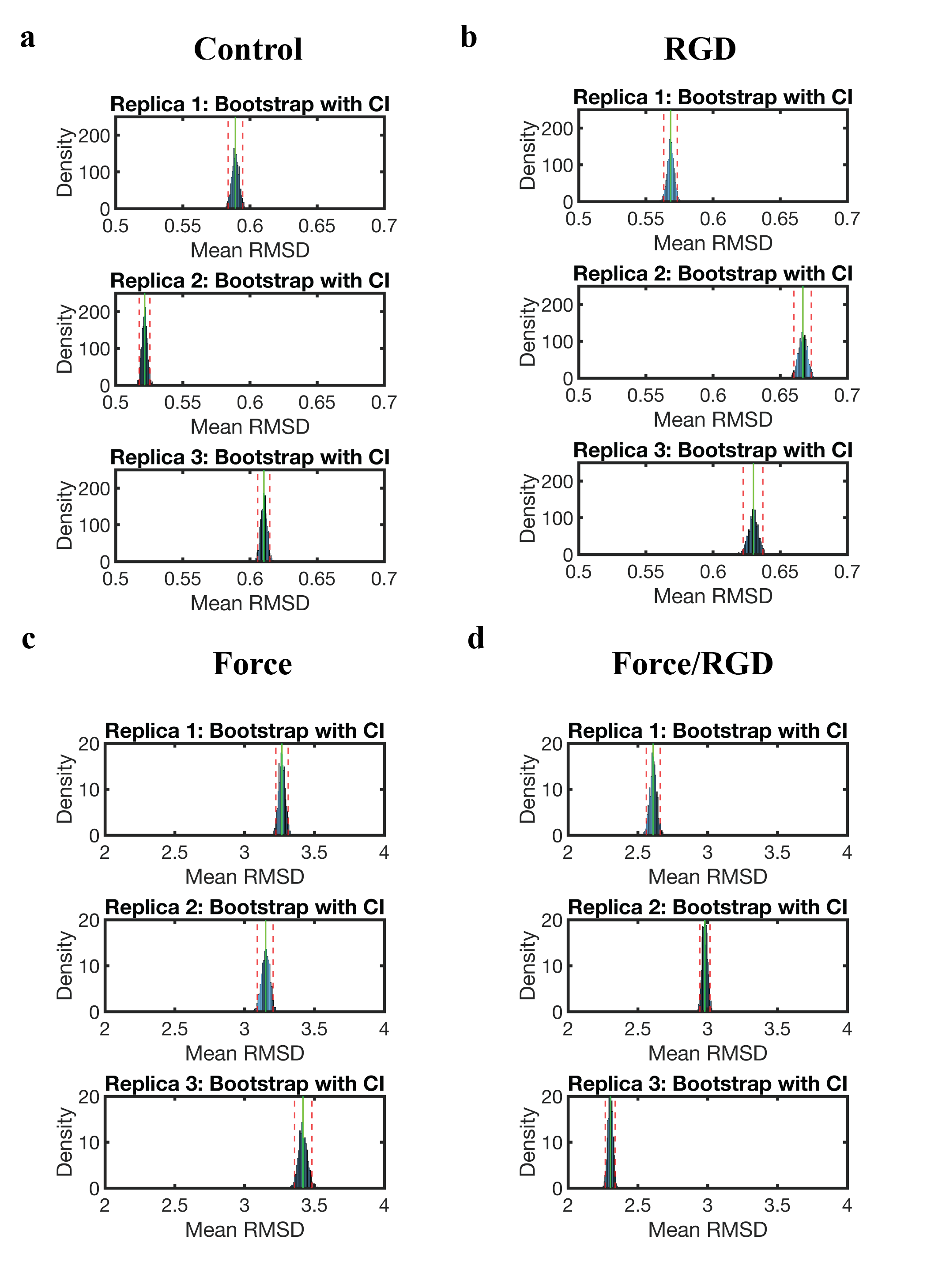

Supplement: Supplementary file 9 — Supplementary Material 9 (PNG401 KB) [file 18_2026_6138_MOESM9_ESM.png]

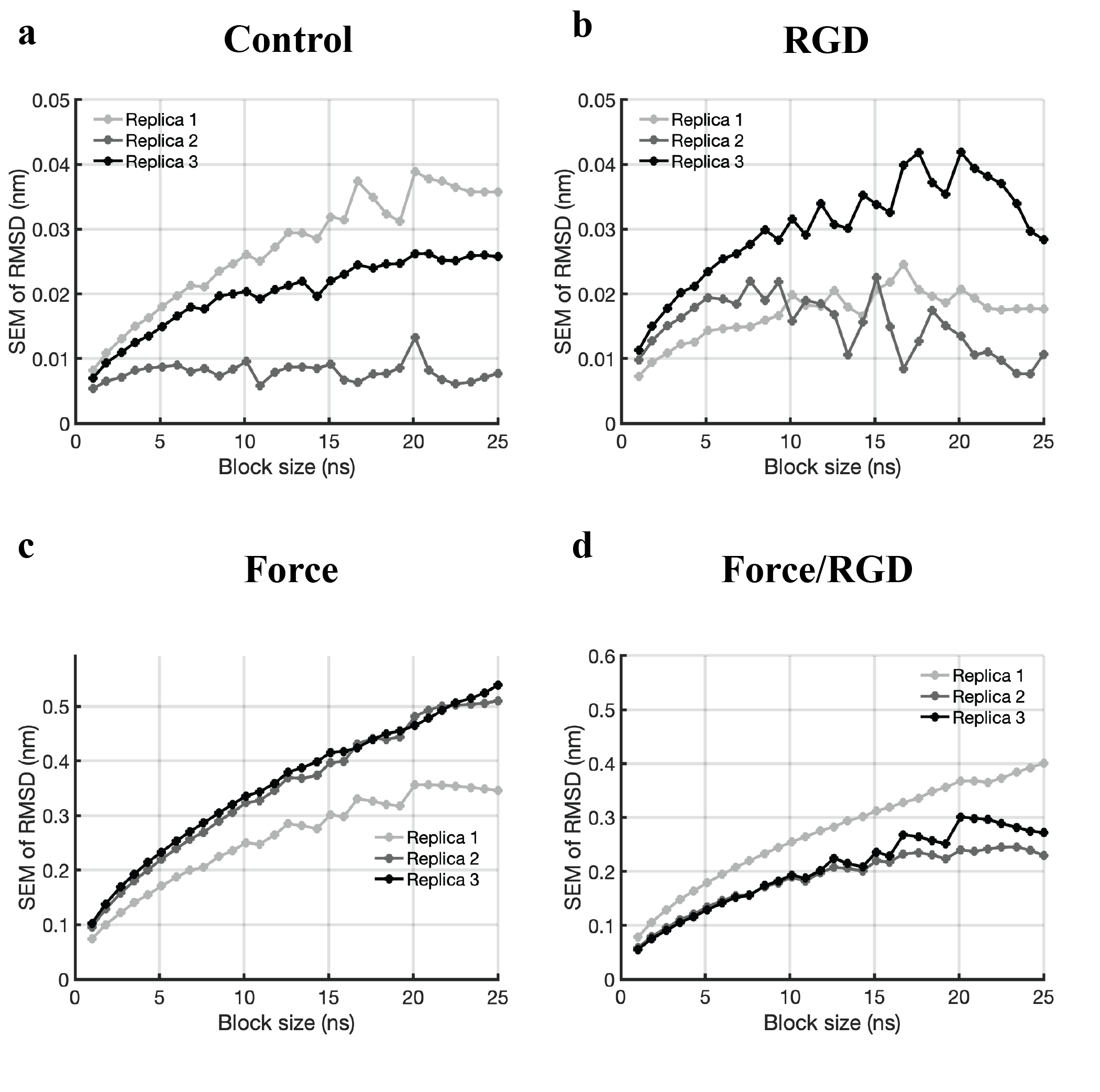

Supplement: Supplementary file 10 — Supplementary Material 10 (PNG238 KB) [file 18_2026_6138_MOESM10_ESM.png]

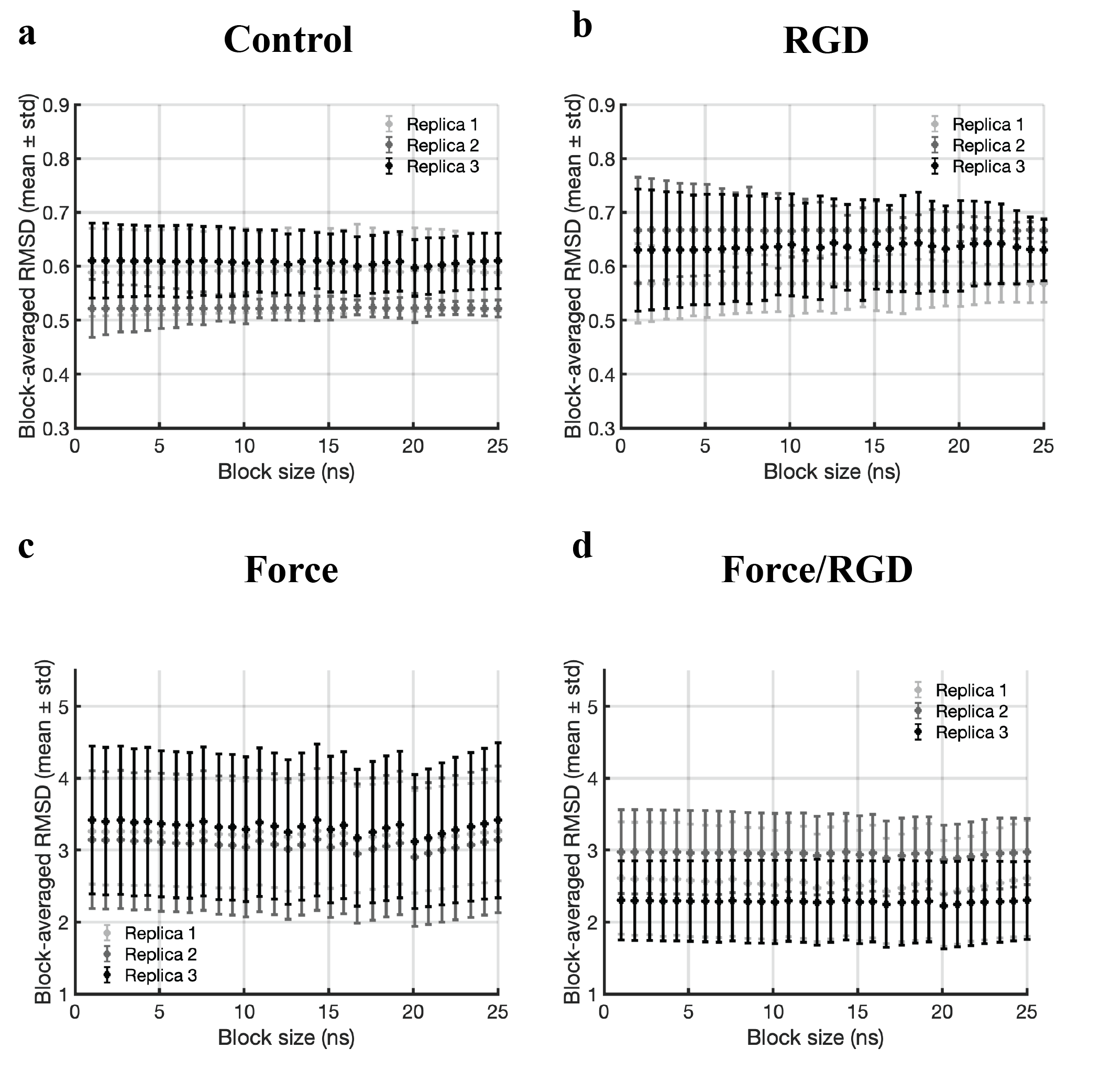

Supplement: Supplementary file 11 — Supplementary Material 11 (PNG224 KB) [file 18_2026_6138_MOESM11_ESM.png]
