# Supplementary material for: All-atom simulations reveal distinct pathways for αIIbβ3 activation by biochemical vs. mechanical cues
Source: Cell Mol Life Sci. 2026 Mar 6;83(1):157. doi: 10.1007/s00018-026-06138-9 (PMC13003053; doi:10.1007/s00018-026-06138-9)
Supplement: Supplementary file 2 [file 18_2026_6138_MOESM12_ESM.docx]

Supplementary information

Membrane-embedded αIIbβ3 simulations

The full-length, all-atom structure of αIIbβ3 was embedded in a lipid bilayer generated using the CHARMM-GUI Membrane Builder. The bilayer was composed of DOPC and DOPS in a 4:1 molar ratio, with the integrin oriented such that the transmembrane αIIb helix was perpendicular to the membrane plane. The final system contained 504 DOPC and 126 DOPS lipids, 1,077 sodium ions, 897 chloride ions, and 323,037 water molecules. The simulation box measured approximately 16.2 × 16.2 × 50 nm, resulting in a total of 1,084,057 atoms. Energy minimization, equilibration under NVT and NPT ensembles, and production simulations followed the same protocol as the corresponding non-membrane systems, using identical temperature control, bond constraints, and treatments of electrostatics and van der Waals interactions. A constant pulling force of 83 pN was applied along the Z axis at the center of mass of the ligand-binding site to mimic physiological extension of the integrin ectodomain.

Principal Component Analysis

Dynamic cross-correlation analysis revealed distinct residue correlations in the presence or absence of mechanical force (Figure 2), suggesting different conformational pathways for integrin activation. To further explore these differences, we performed Principal Component Analysis (PCA) to extract conformational ensembles from the MD trajectories. For the bent conformation, the control condition produced a compact cluster along PC1 (-15 to 22) and PC2 (-23 to 20), reflecting low conformational variability and a stable equilibrium (Figure S9A). RGD binding slightly increased variability along PC1 (-23 to 32) while slightly decreased as comparable to control along PC2 (-14 to 12) (Figure S9B), indicating modest restriction of structural diversity. Force application markedly broadened the distribution along both PC1 (-127 to 85) and PC2 (-47 to 38), consistent with increased flexibility and elevated RMSF values (Figures 3 and S9C). Under Force/RGD, variability narrowed relative to force alone (PC1: -59 to 97; PC2: -46 to 31) but remained greater than in control, suggesting that ligand binding partially counteracts, but does not override, the conformational expansion induced by force (Figure S9D). These results indicate that mechanical stress accelerates conformational changes in bent integrin more effectively than ligand binding alone.

The open conformation exhibited substantial baseline variability along PC1 (-77 to 77) and PC2 (-44 to 63) (Figure S9E), reflecting intrinsic relaxation distinct from the bent state. RGD binding markedly reduced variability (PC1: -61 to 57; PC2: -48 to 36), implying stabilization of the open conformation (Figure S9F). Force alone also restricted conformational space (PC1: -62 to 68; PC2: -62 to 53), particularly along PC1, the axis capturing the greatest variance (Figure S9G). Combined Force/RGD slightly increased the distribution along PC1 but decreased along PC2 (PC1: -77 to 98; PC2: -43 to 54), reinforcing the stabilizing effect of ligand binding under mechanical stress (Figure S9H). Thus, in contrast to the bent state, force and RGD binding stabilize the open conformation, with additive effects when applied together.

PCA supports our structural analyses: the open conformation remains relatively stable with limited fluctuations (Figure 3C, Figure S7), whereas the bent state exhibits enhanced dynamics under force. These findings demonstrate that integrin's structural response to mechanical and ligand cues is conformation-dependent.

RMSD Analysis and Structural Convergence

RMSD values were calculated from trajectory frames aligned to the initial structure to remove global translations and rotations (Figure S1). In equilibrium simulations, all replicas showed an initial increase in Cα RMSD over ~10–20 ns, reaching ~0.5–0.7 nm (Control, Figure S1A; RGD, Figure S1B). In steered MD (SMD) simulations, RMSD increased over longer times (~60 ns) up to 3–5 nm (Force, Figure S1C; Force/RGD, Figure S1D).

Block averaging was applied to assess convergence (Figure S2). RMSD time series were divided into non-overlapping blocks of increasing size (up to 25 ns), and the standard error of the mean (SEM) was computed from block means. Average RMSD remained stable across block sizes—0.5–0.7 nm for Control and RGD, and 2–4 nm for Force and Force/RGD. SEM initially increased with block size due to correlated fluctuations, then plateaued once the block size exceeded the system’s correlation time (Figure S3).

Replica-based bootstrap analysis further confirmed structural stability. Control replicas exhibited low mean RMSD (0.52–0.61 nm; Figure S2A) with modestly overlapping 95% confidence intervals, while RGD replicas showed slightly higher RMSD (0.57–0.67 nm; Figure S2B) but remained converged. Force and Force/RGD conditions showed higher RMSD ranges of 3.2–3.5 nm and 2.3–3.0 nm, respectively (Figure S2C,D). Bootstrap resampling (1,000 iterations) yielded mean RMSDs of 0.574 nm (95% CI: 0.404–0.703 nm) for Control, 0.622 nm (95% CI: 0.43–0.85 nm) for RGD, 3.28 nm (95% CI: 0.87–4.72 nm) for Force, and 2.63 nm (95% CI: 0.77–3.65 nm) for Force/RGD. Narrow confidence intervals under equilibrium conditions indicate stable convergence, while broader intervals under force reflect increased structural variability while capturing representative conformational ensembles.

Overall, these results demonstrate robust structural convergence across all conditions. Equilibrium simulations maintained low RMSD with narrow confidence intervals, indicating stable sampling of basal and ligand-bound conformations. In contrast, SMD simulations exhibited larger RMSD and broader intervals, reflecting force-induced structural deviations while still capturing representative conformational behavior. Combined RMSD trends, block averaging, and bootstrap analyses confirm that our simulations provide reliable and reproducible estimates of integrin dynamics under both equilibrium and mechanically stressed conditions.

Supplementary Figures 

Figure S1. Cα RMSD of bent αIIbβ3 . Cα RMSD versus time is shown for (A) control (no RGD, no force), (B) RGD-bound, (C) force applied, and (D) force applied in the presence of RGD. Individual replicas are shown (three for equilibrium conditions and six for force-applied conditions) over 40 ns of simulation, using 400 frames..

Figure S2. Block-averaged Cα RMSD as a function of block size. Mean square displacement (MSD) values of Cα atoms were computed for each replica and averaged over non-overlapping blocks of increasing size. RMSD versus block size is shown for (A) control, (B) RGD, (C) force, and (D) force/RGD conditions. Stable RMSD values across block sizes indicate structural stability during the simulations..

Figure S3. Convergence of the standard error of the mean (SEM) of Cα RMSD with block size. SEM values were calculated from Cα RMSD averaged over non-overlapping blocks of increasing size to assess statistical convergence. SEM versus block size is shown for (A) control, (B) RGD, (C) force, and (D) force/RGD conditions. The plateau at larger block sizes indicates convergence and effective sampling of independent fluctuations..

Figure S4. Bootstrap distributions of mean Cα RMSD for individual simulation replicas.
Histograms of mean RMSD values were generated using 30 bins and normalized to probability density. Vertical lines indicate the mean RMSD (green) and the 95% confidence interval (red dashed). Panels A and B show equilibrium simulations under control and RGD conditions (three replicas each), whereas panels C and D show force-applied simulations without and with RGD (three replicas each).

.

Figure S5. Bootstrap distributions of pooled Cα RMSD values across replicas. For each condition, 1,000 bootstrap samples were generated by resampling with replacement. RMSD values from all three replicas per condition were pooled to obtain a single distribution, from which the overall mean (green line) and 95% confidence interval (red dashed lines) were calculated. Panels A–D correspond to control, RGD, force, and force/RGD conditions, respectively.

Figure S6. Convergence of Cα root-mean-square fluctuation (RMSF) across time intervals. RMSF values were calculated for each replica over four consecutive 10-ns intervals. Absolute RMSF differences between 0–10 ns vs. 10–20 ns and 20–30 ns vs. 30–40 ns are shown as boxplots for (A) control, (B) RGD, (C) force, and (D) force/RGD conditions. A progressive reduction in RMSF differences indicates stabilization of residue fluctuations and simulation convergence.

Figure S7. Impact of mechanical force and RGD binding on the open conformation of αIIbβ3. (A) Average Cα RMSF under different conditions. (B) Residue-wise Cα RMSD calculated between the initial and final frames. Data were obtained from Cα trajectories of three independent equilibrium and force-applied simulations over 20 ns (200 frames).

Figure S8. Normalized Dynamic cross correlation (nDCC) of residue motions in open αIIbβ3. (A-D) nDCC maps are shown for (A) Control, (B) RGD, (C) Force, and (D) Force/RGD conditions. Data were derived from Cα trajectories of three independent equilibrium and force-applied simulations over 20 ns (200 frames).

Figure S9. Principal component analysis (PCA) of αIIbβ3 integrin dynamics. Projection of simulation trajectories onto the first two principal components (PC1 and PC2) is shown for the bent (A–D) and open (E–H) conformations under control, RGD, force, and force/RGD conditions. All trajectories were projected onto principal components derived from the control simulations. Data were obtained from Cα trajectories of three independent simulations over 100 ns (bent) and 20 ns (open).

Figure S10. Steered molecular dynamics (SMD) simulations of αIIbβ3 integrin under a 20 pN force. Panels A–D correspond to the bent conformation, and panels E–H to the open conformation. (A, E) Time evolution of the average Cα RMSD from three independent replicas. (B, F) Average Cα RMSF. (C, G) Normalized extension of the integrin over time. Conditions are color-coded as control (blue), RGD-bound (cyan), force applied (red), and force applied in the presence of RGD (orange). (D, H) Distribution of correlated motions, shown as the percentage of positively correlated, negatively correlated, and non-correlated residue fluctuations.

Figure S11. Steered molecular dynamics (SMD) simulations of membrane-embedded αIIbβ3 integrin. (A) Time-dependent extension during a 100-ns SMD simulation with αIIbβ3embedded in a DOPC:DOPS lipid bilayer (3:1 molar ratio) and subjected to a constant 83 pN pulling force along the membrane normal (Z axis). (B) Cα RMSD relative to the initial structure. (C) Average Cα RMSF. (D) Normalized dynamic cross-correlation (nDCC) map showing correlated and anti-correlated residue motions. (E) All-atom secondary structure representations of bent αIIbβ3 at 100 ns for the control simulation (left), force applied to the ligand-binding site of αIIbβ3 in the membrane (middle), and force applied to the ligand-binding site of αIIbβ3 in solvent (right).
